# Diet‐induced maternal obesity impacts feto‐placental growth and induces sex‐specific alterations in placental morphology, mitochondrial bioenergetics, dynamics, lipid metabolism and oxidative stress in mice

**DOI:** 10.1111/apha.13795

**Published:** 2022-02-15

**Authors:** Tina Napso, Samantha C. Lean, Minhui Lu, Emily J. Mort, Michelle Desforges, Ali Moghimi, Beverly Bartels, Tatiana El‐Bacha, Abigail L. Fowden, Emily J. Camm, Amanda N. Sferruzzi‐Perri

**Affiliations:** ^1^ Department of Physiology Development and Neuroscience Centre for Trophoblast Research University of Cambridge Cambridge UK; ^2^ Division of Developmental Biology and Medicine Maternal & Fetal Health Research Centre University of Manchester Manchester UK; ^3^ The Children’s Hospital at Westmead Westmead New South Wales Australia; ^4^ Department of Paediatrics Monash University Monash Victoria Australia; ^5^ Present address: Institute of Nutrition Federal University of Rio de Janeiro Rio de Janeiro Brazil; ^6^ Present address: The Ritchie Centre Hudson Institute of Medical Research Clayton Victoria Australia

**Keywords:** fetus, mitochondria, obesity, obesogenic diet, placenta, sex

## Abstract

**Aim:**

The current study investigated the impact of maternal obesity on placental phenotype in relation to fetal growth and sex.

**Methods:**

Female C57BL6/J mice were fed either a diet high in fat and sugar or a standard chow diet, for 6 weeks prior to, and during, pregnancy. At day 19 of gestation, placental morphology and mitochondrial respiration and dynamics were assessed using high‐resolution respirometry, stereology, and molecular analyses.

**Results:**

Diet‐induced maternal obesity increased the rate of small for gestational age fetuses in both sexes, and increased blood glucose concentrations in offspring. Placental weight, surface area, and maternal blood spaces were decreased in both sexes, with reductions in placental trophoblast volume, oxygen diffusing capacity, and an increased barrier to transfer in males only. Despite these morphological changes, placental mitochondrial respiration was unaffected by maternal obesity, although the influence of fetal sex on placental respiratory capacity varied between dietary groups. Moreover, in males, but not females, maternal obesity increased mitochondrial complexes (II and ATP synthase) and fission protein DRP1 abundance. It also reduced phosphorylated AMPK and capacity for lipid synthesis, while increasing indices of oxidative stress, specifically in males. In females only, placental mitochondrial biogenesis and capacity for lipid synthesis, were both enhanced. The abundance of uncoupling protein‐2 was decreased by maternal obesity in both fetal sexes.

**Conclusion:**

Maternal obesity exerts sex‐dependent changes in placental phenotype in association with alterations in fetal growth and substrate supply. These findings may inform the design of personalized lifestyle interventions or therapies for obese pregnant women.

## 1 INTRODUCTION

As obesity increases worldwide, the proportion of women entering pregnancy overweight or obese is rising rapidly.^1^ In the UK, more than half of pregnant women (52.7%) have a body mass index (BMI) above the normal healthy range.^2^ This is concerning as pre‐pregnancy obesity increases the risk of pregnancy complications, such as miscarriage, gestational diabetes mellitus (GDM), pre‐eclampsia, pre‐term birth and still‐birth.^3^ Maternal obesity also increases the spectrum of birthweights, with increased rates of macrosomia, intrauterine growth restriction and of both large and small for gestational age infants.^1^, ^3^, ^4^ Abnormal birth weight increases the risk of poor neonatal viability, neurodevelopmental and immune disorders during childhood, and of obesity, type‐2 diabetes, heart disease and neurocognitive impairments in adulthood.^1^ These adverse effects of maternal obesity have profound public health implications, and highlight the urgency of establishing the mechanisms involved.

As the interface between the mother and fetus, the placenta has a central role in determining growth and development of the fetus and, hence, its life‐long health.^5^ The placenta affects the fetal supply of nutrients and the secretion of hormones and growth factors that adapt maternal metabolism to support the pregnancy.^6^, ^7^ The placenta must also grow and adapt morphologically and functionally in response to the developmental increase in fetal nutrient demands and to environmental changes in maternal resource availability.^8^ Maternal obesity is known to alter the growth, nutrient transport and endocrine function of the placenta from the earliest stages of development in rodents and other non‐human species fed obesogenic diets.^9^, ^10^ Similar phenotypical changes have also been observed in the placenta of obese pregnant women at term.^11^ Maternal obesity also leads to abnormalities in uterine blood flow and to alterations in the maternal endocrine and metabolic environment associated with dyslipidaemia and hyperglycaemia.^12^, ^13^, ^14^ Furthermore, several recent studies have reported that the effects of maternal obesity on placental phenotype are sex‐linked,^15^, ^16^, ^17^ although, the mechanisms underlying this sexual dimorphism remain poorly understood. Consequently, maternal obesity may compromise normal fetal growth via changes in the maternal supply of nutrients and oxygen for placental transport and/or in the placental phenotype itself in a sex‐linked manner.

Given the high energy demands of the placenta, obesity‐induced changes in placental phenotype may stem, in part, from alterations in placental mitochondrial function. In adult metabolic tissues, mitochondria are dynamic organelles that are responsive to energy demands and environmental cues. In the placenta, they are the main source of energy (ATP) for growth, nutrient transport and endocrine function.^18^ Mitochondria produce ATP by oxidative phosphorylation (OXPHOS) involving a sequence of electron transfer reactions at five protein complexes in the inner mitochondrial membrane, collectively termed the electron transfer system (ETS).^19^ Reducing equivalents generated from the tricarboxylic acid cycle and the β‐oxidation of fatty acids serve as electron sources for complexes I and II of the ETS.^18^ As a natural by‐product of the ETS, mitochondria also produce reactive oxygen species (ROS), which are important in cell signalling. However, excessive ROS production can lead to oxidative stress, impaired mitochondrial respiratory efficiency, and apoptosis. Mitochondrial dysfunction associated with obesity has been studied predominantly in highly metabolic tissues in adult animals (eg adipose, heart, liver, skeletal muscle). Emerging evidence suggests that pre‐pregnancy maternal obesity adversely affects placental mitochondria, with abnormal morphology and decreased abundance, reductions in ETS complex and ATP synthase content, consistent with compromised OXPHOS.^20^, ^21^, ^22^, ^23^ In human term placenta, basal, maximal and ADP‐coupled rates of mitochondrial O_2_ utilisation are all inversely correlated to maternal body mass index (BMI).^21^ The compromised ETS in placental mitochondria also leads to enhanced placental production of ROS.^22^, ^23^ Of these studies, only two have considered whether placental mitochondrial function is sex‐linked in either normal or obese pregnancies.^21^, ^24^ Indeed, the links between fetal growth, placental morphology and placental mitochondrial function, and the influence of fetal sex, remain unclear.

Here, we used a novel diet‐induced obesity mouse model in which female C57BL6/J female mice were fed a control or a high‐fat, high‐sugar diet for 6 weeks before and during pregnancy. At day 19 of gestation (term 20.5 days), placentas were collected. Using high‐resolution respirometry, stereology, and molecular analyses, mitochondrial respiratory capacity, mitochondrial regulator protein abundance (ETS, lipid metabolism, dynamic regulators and stress signalling), and oxidative stress levels of the placenta were determined, in relation to placental morphology and fetal growth in female and male offspring. We hypothesized that maternal obesity would compromise fetal growth in association with changes in placental morphology, mitochondrial function and oxidative stress in a sex‐specific manner.

## 2 RESULTS

### 2.1 Diet‐induced obesity moderately alters maternal endocrine and metabolic state in pregnancy

Dams fed the obesogenic diet for 6 weeks prior to mating were significantly heavier (~+10%) at day 1 of pregnancy compared to dams fed the standard control diet (Table 1). There was no effect of diet on plugging rate (mean ± SEM days: control diet 2.6 ± 0.4, obese diet 3.4 ± 0.6, n = 9/group). At day 19 of pregnancy (term day 20.5), the obese diet had no effect on total body weight of the dams, however, hysterectomised weight was significantly reduced (~−10%; Table 1). Dams fed the obesogenic diet also showed ~25% reduction in gestational weight gain (Table 1). Despite these observations, dams fed the obesogenic diet showed ~70% increase in adiposity with a concomitant decrease in lean mass, compared to control dams at day 19 of pregnancy (Table 1), which was measured by dual energy X‐ray absorptiometry (DEXA) scanning. As the percent adiposity of the dams fed the obesogenic diet was greater than three‐times the standard deviation of the control mean value (a reported proxy for obesity in mice^25^), the diet was successful in inducing obesity in our dams. Hence, this experimental group was labelled as diet‐induced obesity/diet‐induced obese dams subsequently.

**TABLE 1:** Characteristics of control dams and the impact of diet‐induced obesity

	Control diet	Obese diet	*P* value
Weight day 1 (g)	23.6 ± 0.6	25.9 ± 0.7	.**019**
Weight day 19 (g)	41.8 ± 1.2	39.4 ± 0.6	.111
Hysterectomised weight (g)	28.4 ± 0.8	25.7 ± 0.6	.**019**
Pregnancy weight gain of maternal tissues (g)	18.2 ± 0.7	13.5 ± 0.5	.**001**
% Fat mass	20.2 ± 0.7	34.0 ± 1.8	**<.001**
% Lean mass	79.9 ± 0.7	66.1 ± 1.8	**<.001**
Glucose (mmol/L)	9.3 ± 1.0	12.1 ± 0.7	.**034**
Insulin (μg/L)	0.33 ± 0.05	0.22 ± 0.33	.070
Leptin (ng/mL)	19.10 ± 0.13	20.48 ± 0.23	.962
Adiponectin (μg/mL)	23.61 ± 0.67	25.22 ± 0.56	.085
Triglycerides (mmol/L)	0.92 ± 0.11	0.38 ± 0.06	.**001**
NEFA (μmol/L)	370.0 ± 73.5	205.8 ± 32.9	.059
GH (ng/mL)	20.23 ± 5.00	37.88 ± 5.40	.053
Prolactin (ng/mL)	143.64 ± 23.48	185.88 ± 30.22	.286
TSH (pg/mL)	327.9 ± 77.4	463.5 ± 32.2	.052
LH (pg/mL)	268.0 ± 59.0	354.9 ± 64.2	.**008**
Progesterone (ng/mL)	11.1 ± 3.0	11.8 ± 3.4	.736
FSH (pg/mL)	1158.2 ± 431.9	634.1 ± 54.7	.847
Litter size	8.2 ± 0.3	8.7 ± 0.6	.483
% Females	48.1 ± 5.0	41.4 ± 5.3	.370
Non‐viable pups per litter (%)	3.9 ± 2.5	8.3 ± 4.8	.752

Note

Data are from 8‐9 dams per group and presented as mean ± SEM at day 19 of pregnancy. Gestational weight gain was calculated as the body weight on day 19 of pregnancy minus weight on day 1 of pregnancy. The percentage of viable pups was calculated as the proportion of live pups minus the number of reabsorptions per litter. Significant difference between control and obese dams (*P* < .05, unpaired *t* test or Mann Whitney Rank Sum Test) indicated in bold.

Diet‐induced maternal obesity was associated with significantly altered circulating concentrations of maternal glucose (~+30%), triglycerides (~‐60%) and luteinizing hormone (LH) (~+30%) in late pregnancy (Table 1). Plasma thyroid stimulating hormone (TSH), insulin, non‐esterified free fatty acids (NEFA), leptin, adiponectin, progesterone and other pituitary hormone concentrations (growth hormone [GH], prolactin, follicle stimulating hormone [FSH]) were not significantly different between control and diet‐induced obese dams in late gestation (Table 1). Thus, diet‐induced maternal obesity moderately alters maternal endocrine and metabolic profile in late pregnancy.

### 2.2 Diet‐induced maternal obesity alters fetal growth and glycemia, as well as placental weight in late gestation

**FIGURE 1:**
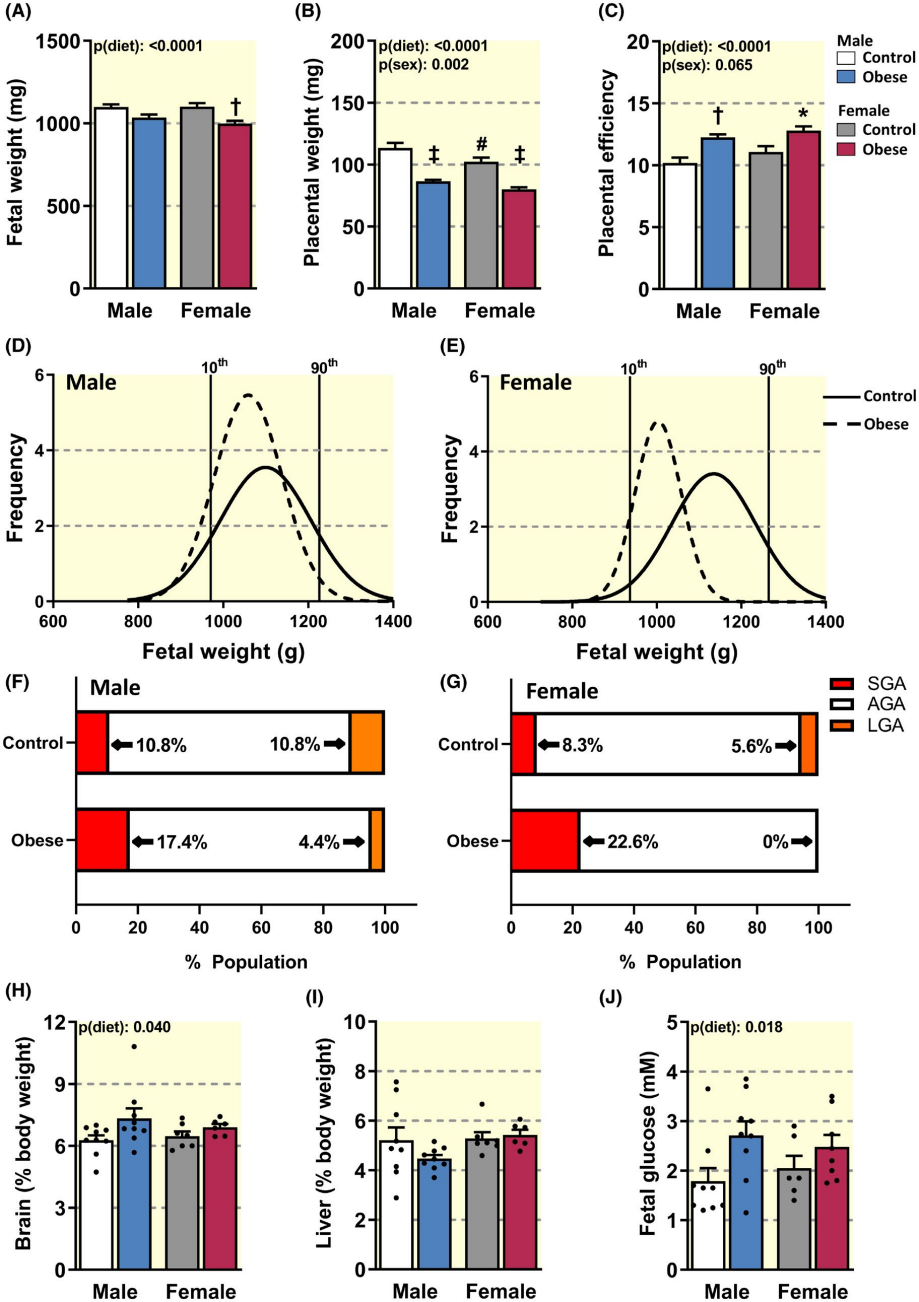
Fetal and placental weights in control dams and the impact of diet‐induced obesity. Fetal weight (A), placental weight (B) and placental efficiency (c) for male and female fetuses, fetal weight frequency distribution curves for male (D) and female fetuses (E), percentage of fetuses classified as small for gestational age (SGA), appropriate for gestational age (AGA) and large for gestational age (LGA) for male (F) and female (G) offspring and fetal fractional brain and liver weights (H and I), and glucose concentrations (J) for males and females. D and E, are based on z‐score centiles, where vertical solid lines represent the 10th and 90th centile (z‐scores −1.282 and 1.282 respectively) of the control curve for male fetuses (970.7 and 1226.0 mg, respectively) and female fetuses (936.8 and 1264.9 mg, respectively). Data in A‐C and H‐J are displayed as mean + SEM, with individual values shown in H‐J from 7‐9 litters per group and were analysed by two‐way ANOVA mixed linear model (fetal sex [p(sex)], diet‐induced maternal obesity [p(diet)], interaction [p(int)]) with Tukey post hoc pairwise comparisons. Significant difference between control and diet‐induced obese dams for fetuses of the same sex (*P < .05, ^†^ *P* < .001, ^‡^ *P* < .0001, pairwise comparison). ^#^Significant difference between male and female fetuses within the same maternal dietary group (^#^ *P* < .05, pairwise comparison)

Overall, diet‐induced maternal obesity reduced fetal weight in late gestation, with pairwise comparisons revealing female offspring were 9% lighter in obese versus control dams (6% reduction in males, *P* > .05; Figure 1A). Placental weight was also reduced by diet‐induced maternal obesity, by 24% and 22% in males and females, respectively (Figure 1B). In control dams, placental weight was significantly greater for male compared to female fetuses, but not in obese dams (Figure 1B). As the decrement in placental weight was more severe than that of the fetus, placental efficiency was increased by 20% and 16% in male and female offspring of diet‐induced obese dams, respectively (Figure 1C). However, despite this increase in placental efficiency, the distribution curve for fetal weights was shifted to the left for both male and female offspring of diet‐induced obese dams, with ~60% and ~170% more fetuses falling below the 10th centile (small for gestational age, SGA), respectively and ~60% fewer or 0% above the 90th centile (large for gestational age, LGA), respectively when compared to control fetal weight (Figure 1D‐G). There was also a narrowing of the range of weights in female, but not male fetuses, in diet‐induced dams. There was an overall effect of diet‐induced maternal obesity to increase relative fetal brain weight, but had no effect on the fractional weight of the fetal liver (Figure 1H,I). However, overall, blood glucose concentrations were elevated in offspring from obese dams. There was no effect of diet‐induced maternal obesity on litter size, fetal viability or the sex ratio within the litter (Table 1). Thus, diet‐induced maternal obesity negatively affects both fetal and placental growth in late gestation.

### 2.3 Diet‐induced maternal obesity alters placental morphology in a sex‐dependent manner

**FIGURE 2:**
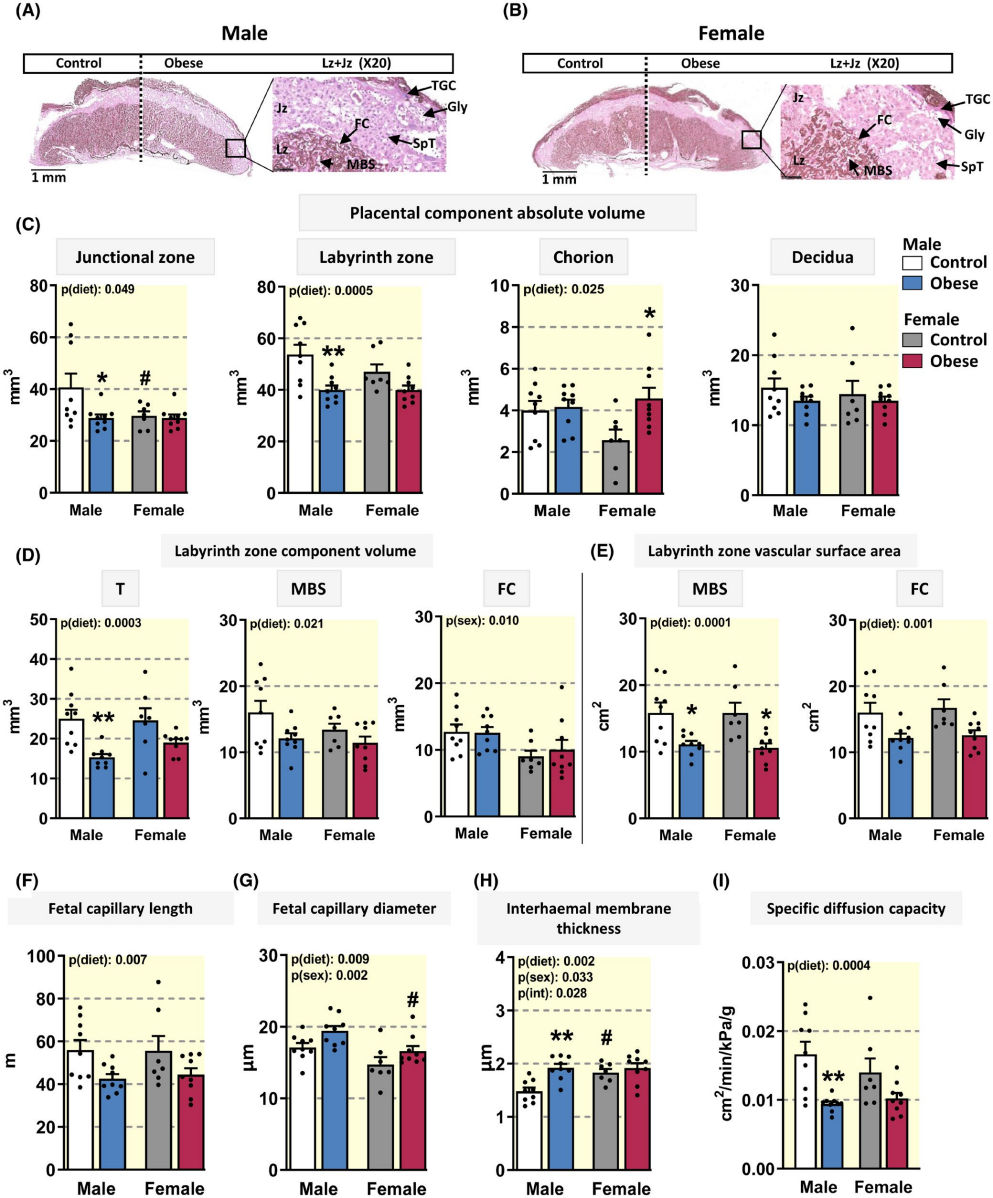
Placental morphology in control dams and the impact of diet‐induced obesity. Representative images of haematoxylin and eosin‐stained male (A) and female placentas (B) from control and diet‐induced obese dams. Scale bar: 1 mm with higher magnification showing the structure of the labyrinth zone, scale bar: 100 µm. Placental region volumes (C), labyrinth zone component volumes (D), vascular surface areas (E), fetal capillary length (F), fetal capillary diameter (G), thickness of the interhaemal membrane (H) and specific diffusion capacity (I) for male and female fetuses. Data in C‐I are displayed as individual values with mean +SEM and 1 fetus per sex in each of 7‐9 litters per group and were analysed by two‐way ANOVA (fetal sex [p(sex)], diet‐induced maternal obesity [p(diet)], interaction [p(int)]) with Tukey post hoc pairwise comparisons. Significant difference between control and diet‐induced obese dams for fetuses of the same sex (*P < .05, **P < .01, pairwise comparison). ^#^Significant difference between male and female fetuses within the same maternal dietary group (^#^ *P* < .05, pairwise comparison). FC, fetal capillaries; Gly, glycogen cells; Jz, junctional zone; Lz, labyrinth zone; MBS, maternal blood spaces; SpT, spongiotrophoblast; T, trophoblast; TGC, giant cells

Reductions in feto‐placental weight in obese pregnancies were accompanied by changes in placental morphology, assessed by histological staining and unbiased stereology (Figure 2 and Table 2). The estimated volumes of the placental junctional zone (synthesis of endocrine mediators; Jz) and labyrinth zone (functions in substrate exchange; Lz) were reduced by diet‐induced maternal obesity, with a significant effect observed in males (−30% and −26%, respectively), but not females (Figure 2A‐C). Compared to females, male placentas had a significantly greater Jz volume in control but not diet‐induced obese dams. There was an overall effect of diet‐induced maternal obesity to increase the volume of the chorion, and post‐hoc analysis revealed that this effect was significant only in female offspring (+177%; Figure 2C). However, the volume of the maternal decidua was not altered significantly by diet‐induced maternal obesity in either sex.

**TABLE 2:** The morphology of the placental junctional zone in control dams and the impact of diet‐induced obesity

	Male fetuses		Female fetuses		p(diet)	p(sex)	p(int)
	Control	Obese diet	Control	Obese diet			
*Estimated volume (mg)*							
Gly	5.64 ± 1.37	4.66 ± 0.68	3.90 ± 1.09	4.58 ± 0.46	0.875	0.352	0.400
SpT	33.83 ± 3.92	23.25 ± 1.13	26.99 ± 1.43	22.03 ± 0.85	**0.002**	0.083	0.221
TGC	1.19 ± 0.28	0.97 ± 0.17	0.50 ± 0.12	0.86 ± 0.19	0.744	0.059	0.176

Note

Data are from fetuses per sex across 8‐9 different litters per treatment and presented as mean ± SEM values. Statistical significance for the comparison of control and obese data with diet and sex as factors plus their interaction (*P* < .05, two‐way ANOVA and post hoc Tukey test). Effect of fetal sex [p(sex)], maternal obesity [p(diet)], interaction [p(int)] are shown, with statisitcal significance indicated in bold. Gly, glycogen cell; SpT, spongiotrophoblast cell; TGC, trophoblast giant cell.

In the placental Lz, diet‐induced maternal obesity was associated with a decrease in the volume of trophoblast, an effect that was significant in males (−39%), but not females (Figure 2D). Diet‐induced maternal obesity reduced the volume and surface area of maternal blood spaces by 25% and 30% in males and by 14% and 33% females, respectively (Figure 2D,E). Similar negative effects of maternal diet‐induced obesity were observed for the surface area of fetal capillaries in the placental Lz of males and females (−24% and −13%, respectively; Figure 2D,E). Moreover, in the placental Lz, diet‐induced maternal obesity reduced fetal capillary length by 24% and 30% in males and females, respectively, and increased fetal capillary diameter by 13%‐14% in both sexes (Figure 2F,G). When dietary groups were combined, fetal capillary volume and diameter in the placental Lz were greater in males versus females. Pairwise comparisons showed that fetal capillary diameter in males was only significantly greater in diet‐induced obese dams. The thickness of trophoblast barrier to diffusion was increased by 35% specifically in the male placenta of diet‐induced obese dams, relative to the controls (Figure 2H). Furthermore, Lz barrier thickness was less in males versus females in control, but not diet‐induced obese dams. Finally, the specific diffusing capacity of the placenta for diffusion of molecules including oxygen was diminished in diet‐induced obese dams, and this effect was significant for males of diet‐induced obese dams (Figure 2I).

In the Jz, the estimated volume of spongiotrophoblast cells was reduced by 32% and 18% in males and females, respectively, however, glycogen cells and giant cells remained unaltered regardless of sex, in obese versus control dams (Table 2). Taken together, these data indicate that diet‐induced maternal obesity affects placental morphology, with impacts on the placental transport zone that appear to be sex‐dependant. Furthermore, the changes in placental structure observed in offspring from diet‐induced obese dams are in line with the decreased fetal growth observed in this cohort.

### 2.4 Placental zone respiratory capacity differs depending on fetal sex and this is modified by maternal diet‐induced obesity

**FIGURE 3:**
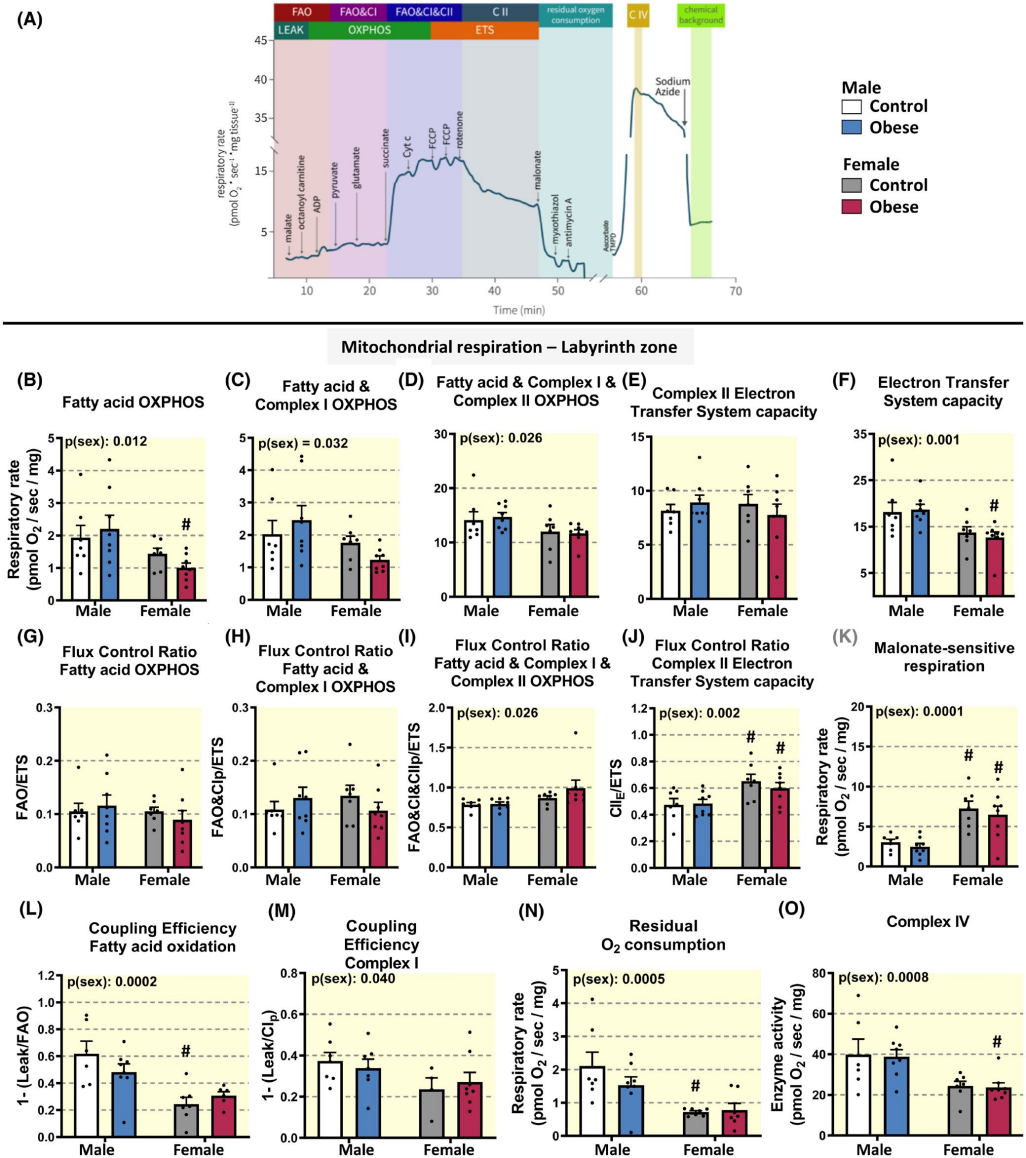
Placental labyrinth zone respiratory capacity in control dams and the impact of diet‐induced obesity. Representative high‐resolution respirometry trace showing the sequential steps in the substrate‐uncoupler‐inhibitors titration (SUIT) protocol and the respective respiratory states (A); mitochondrial respiration in the placental labyrinth zone (B‐K). B‐D, correspond to fatty acid, complex I and complex II substrate‐driven respiration; (E) a corresponds to complex II‐driven oxygen consumption after addition of rotenone; (F) corresponds to maximal electron transfer system (ETS) capacity after addition of FCCP; (G‐J) correspond to oxygen consumption for the respective substrates relative to ETS capacity; (K) corresponds to the fraction of oxygen consumption that was inhibited by malonate. Data in L and M correspond to coupling efficiency after addition of the stated respiratory substrates calculated as 1‐ (Leak/Coupled respiration) where Leak is the oxygen consumption after addition of malate and octanoylcarnitine; (N) correspond to oxygen consumption not related to mitochondrial complexes, calculated after addition malonate, antimycin A and myxothiazol. O, corresponds to the enzymatic assay of complex IV activity, measured in the same run as the respiratory states using the artificial substrate TMPD and ascorbate. Data are displayed as individual values with mean + SEM with 1 fetus per sex in each of 7‐9 litters per group and were analysed by two‐way ANOVA (fetal sex [p(sex)], diet‐induced maternal obesity [p(diet)], interaction [p(int)]) with Tukey post hoc pairwise comparisons. No significant differences between control and diet‐induced obese dams by pairwise comparisons. ^#^Significant difference between male and female fetuses within the same maternal dietary group (^#^ *P* < .05, pairwise comparison)

Alterations in placental morphology and fetal growth in pregnancies complicated by maternal obesity could compromise placental mitochondrial function, and potentially underlie their increased susceptibility of these pregnancies to fetal demise and to developmental programming. To examine this possibility, mitochondrial OXPHOS was measured using high‐resolution respirometry to assess respiratory capacities and respiratory coupling of single and combined mitochondrial ETS pathways in micro‐dissected placental Lz and Jz samples (Figure 3A).

#### 2.4.1 Placental labyrinth zone

There was no effect of maternal diet‐induced obesity on placental Lz respiratory capacity in leak state (leak respiration: mean ± SEM pmol O_2_/sec/mg: control male 0.82 ± 0.16, obese diet male 1.02 ± 0.14, control female 1.09 ± 0.16, and obese‐diet female 1.81 ± 0.07, for obese‐diet female, n = 7‐9). There was also no significant effect of diet‐induced maternal obesity on placental Lz respiratory capacity in OXPHOS state (Figure 3B‐D; note fatty acid oxidation [FAO]_&_ complex I [CI] _p_ appeared reduced specifically in females by *t* test with *P* = .04). However, fetal sex had an overall effect on several measures of placental Lz respirometry. Overall, there was a significant effect of sex on OXPHOS capacity supported by substrates for FAO, CI_P_, and CI_P_ & complex II (CII)_P_, with females presenting significantly lower rates compared to males (Figure 3B‐D). Additionally, when data were split into dietary groups, there was a significant 54% decrease in FAO in female versus male offspring from obese dams (Figure 3B). However, after addition of rotenone, sex differences were no longer observed, regardless of the maternal dietary group (Figure 3E). Overall, maximal ETS capacity induced by the uncoupler trifluoromethoxy carbonyl‐cyanide phenylhydrazone (FCCP), confirmed that the placental Lz of females presented lower rates compared to males (Figure 3F), with pairwise comparisons revealing that maximum respiratory capacity was 32% lower in females compared to males in diet‐induced obese but not control dams. ETS capacity was used as the common (internal) reference state to obtain information of the contribution of all respiratory states normalised to the maximum oxygen consumption rate, after addition of FCCP (flux control ratios; FCRs). This approach provides a powerful normalization of flux and allows the evaluation of changes in oxidative phosphorylation regulation. FCRs for FAO and CI _p_ ‐linked substrates were similar among groups (Figure 3G,H). The FCR for FAO&CI _p_ &CII _p_ was overall greater in females compared to males (Figure 3I). The FCR for succinate‐driven respiration in the electron transfer respiratory capacity state (CII _ets_) was also significantly higher in the Lz of females compared to males however, with pairwise comparisons showing that this was irrespective of diet (Figure 3J). Overall, the fraction of malonate‐sensitive respiration was 2.5‐times higher in females compared to males (Figure 3K).

Coupling efficiencies for FAO (Figure 3L) and CI‐linked (Figure 3M) substrates were lower in Lz samples from females compared to males, with pairwise comparisons revealing that the former was significant only in control dams. Residual oxygen consumption was also 2.5‐fold higher in the placental Lz of males compared to females; an effect that was significant in control dams (Figure 3N). Finally, lower rates for complex IV in females compared to males were observed (Figure 3O), and this difference (−40%) was significant in diet‐induced obese dams.

#### 2.4.2 Placental junctional zone

There were no differences in leak respiration in the placental Jz (mean ± SEM pmol O_2_/sec/mg: control male 1.42 ± 0.21, obese diet male 1.32 ± 0.27, control female 1.09 ± 0.16, and obese‐diet female 1.27 ± 0.19, n = 7‐9). Additionally, respiration with substrates (malate and octanoyl carnitine) for FAO and the NADH‐pathway via CI (pyruvate and glutamate) was similar among groups (Figure S1A,B). Upon addition of succinate (substrate for CII), maximal OXPHOS capacity was stimulated (FAO_&_CI _p_ &CII _p_ ; Figure 3A), with ~25% greater respiration in the placental Jz of females than males (Figure S1C). No differences were observed on maximal electron transfer capacity upon addition of FCCP, nor electron transfer through the succinate‐pathway (after rotenone) with either diet‐induced maternal obesity or fetal sex (Figure S1D,E). The FCR for FAO and for CI‐linked substrates were similar among groups (Figure S1F,G). However, regardless of dietary group, overall relative rates for FAO&CI _p_ &CII _p_ OXPHOS in the placental Jz were higher in females than males (Figure S1H). FCR of complex II‐linked respiration was also similar among groups (Figure S1I). Complex II‐linked respiration was also evaluated after addition of malonate, a competitive inhibitor of this complex, with no differences observed between the groups (Figure S1J).

Coupling efficiency for FAO was calculated and no differences were observed in the placental Jz with diet‐induced maternal obesity (Figure S1K). Residual oxygen consumption was measured after addition of malonate, myxothiazol, and antimycin A (inhibitors of complex III; Figure 3A), and was found to be 1.8‐fold greater in the placental Jz of males compared to females (Figure S1L). Lastly, complex IV showed no differences in activity with diet‐induced maternal obesity or fetal sex (Figure S1M).

Collectively, these data suggest that oxidative phosphorylation capacity differs depending on fetal sex, particularly in the placental Lz, and this is modified by maternal diet‐induced obesity.

### 2.5 Diet‐induced maternal obesity alters the abundance of specific mitochondrial‐related proteins in the placental labyrinth in a manner dependent on fetal sex

**FIGURE 4:**
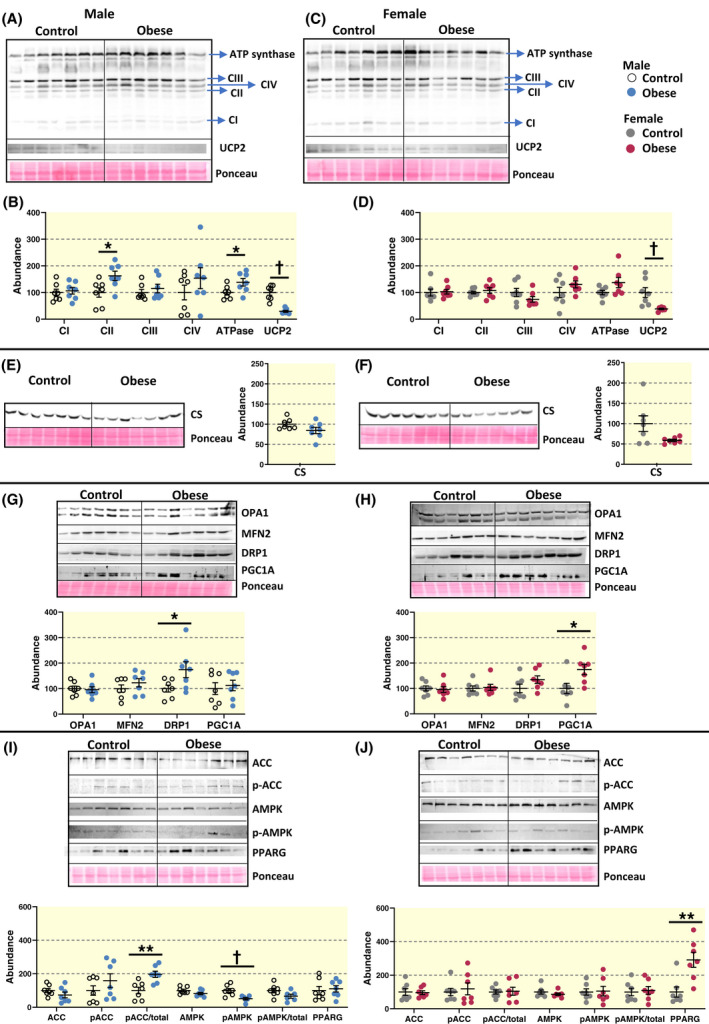
Abundance of mitochondrial‐related proteins in control dams and the impact of diet‐induced obesity. Relative protein abundance of mitochondrial complexes (CI‐CIV and ATP synthase [ATPase]), as well as uncoupling protein‐2 (UCP2) in males (A, B) and females (C, D), citrate synthase (CS) in males (E) and females (F), fission, fusion and biogenesis related proteins in males (G) and females (H), and lipid and energy metabolism proteins (total and phosphorylated acetyl‐CoA carboxylase [ACC and p‐ACC, respectively], total and phosphorylated 5' AMP‐activated protein kinase [AMPK and p‐AMPK], and peroxisome proliferator activated receptor‐γ [PPARG]) in males (I) and females (J). Immunoblot images, as well as ponceau‐S‐stained membrane are shown along with quantification data displayed as individual data points and means ± SEM. Data are from 6‐7 litters per group (note one value for obese male p‐AMPK identified as a statistical outlier and not included for the quantification for p‐AMPK or p‐AMPK/total), with 1 pup per sex in each litter and were analysed by unpaired *t* test or Mann‐Whitney Rank Sum Test (*P < .05, **P < .01, ^†^ *P* < .001)

To investigate the effects of diet‐induced maternal obesity on mitochondrial bioenergetics and dynamics, the abundance of mitochondrial regulatory proteins of the ETS were examined (Figure 4). The placental Lz is critical for supplying O_2_ and nutrients, and given the profound morphological defects observed in the Lz in obese dams, this region was the focus of the molecular work. The abundances of CII and ATP synthase were increased by 63% and 38%, respectively, by diet‐induced maternal obesity in male, but not female offspring (Figure 4A‐D). Uncoupling protein‐2 (UCP2), which uncouples oxygen consumption from ATP synthesis, was decreased by ~70% and ~55% by diet‐induced maternal obesity in the placental Lz of males and females, respectively. Furthermore, the abundance of dynamin related protein (DRP1), which promotes mitochondrial fission, was increased by 74% in the placental Lz of male fetuses, but not females, in diet‐induced obese dams (Figure 4G,H). However, peroxisome proliferator‐activated receptor gamma coactivator 1‐α (PGC1A), which regulates mitochondrial biogenesis, was up‐regulated by 74% in the Lz of females, but not males by maternal obesity. In contrast, the abundance of citrate synthase, a proxy for mitochondrial content, and other ETS complexes and mitochondrial fusion‐fission proteins in the Lz, were unaffected by diet‐induced maternal obesity in either fetal sex (Figure 4). Hence, diet‐induced maternal obesity affects the abundance of specific mitochondrial‐related proteins in the placental Lz in a sex‐linked manner, which likely reflect compensatory mechanisms to maintain Lz mitochondrial respiration in obese pregnancies.

### 2.6 Diet‐induced maternal obesity alters the abundance of mitochondrial‐related lipid metabolic proteins in the placental labyrinth in a sex‐dependent fashion

The level of inactivated acetyl‐CoA carboxylase (ACC; represented as phosphorylated [inactivated] ACC to total ACC), which would catalyze the formation of malonyl‐CoA, the rate‐limiting step in fatty acid synthesis, was increased by 98% in the placental Lz of male fetuses, whereas the abundance of peroxisome proliferator activated receptor‐γ (PPARG), which would increase lipid synthesis, was elevated by 290% only in females from diet‐induced obese dams (Figure 4I,J). The Lz abundance of phosphorylated, active 5' AMP‐activated protein kinase (AMPK), a sensor of cellular energy and key regulator of ACC, was reduced by 49% by diet‐induced maternal obesity in males, but not females (Figure 4I,J). Hence, fetal sex has an important role in determining the effect of diet‐induced maternal obesity on the abundance of mitochondrial‐related lipid handling proteins in the placental Lz, consistent with the sex‐linked differences in fetal growth observed in the obese dams.

### 2.7 Diet‐induced maternal obesity effects oxidative stress signalling pathways and the levels of calcification in the placental labyrinth zone a fashion that partly depends on fetal sex

**FIGURE 5:**
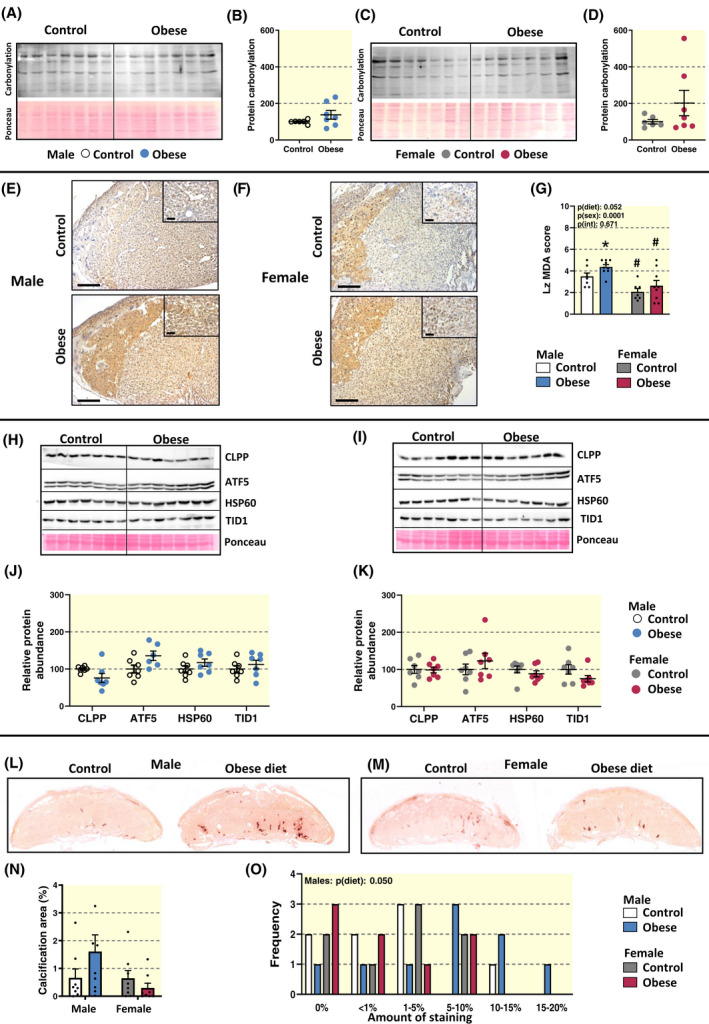
Levels of oxidative stress and calcification in the placental labyrinth zone in control dams and the impact of diet‐induced obesity. Immunoblot images and quantification of oxidised proteins in the labyrinth zone for males (A, B) and females (C, D) expressed relative to controls. Note one value for male and one value for female from control dams shown in the immunoblot was identified as a statistical outlier and not included for the quantification. Lipid peroxidation was indicated by MDA staining and a representative image of the placenta for male and female fetuses (E and F, respectively) and its quantification using a 5‐point scale/scoring system in the labyrinth zone are shown (G). Scale bars in E and F on the lower magnification images are 250 µm and on the higher magnification images are 50 µm. Immunoblot images and quantification of mitochondrial unfolded protein response proteins in the labyrinth of males (H, J) and females (I, K). Representative ponceau‐S‐stained membrane are shown. Note one value for the obese group for ATF5 was identified as a statistical outlier and not included for quantification. Calcification was indicated by alizarin staining and a representative image of the placenta for male and female fetuses (L and M, respectively) and its quantification in the labyrinth zone are shown (N, O). Data are from 7 litters per group, with 1 pup per sex in each litter and displayed as individual data points and means ± SEM. Data in C, D, K and N were analysed by two‐way ANOVA (fetal sex [p(sex)], diet‐induced maternal obesity [p(diet)], interaction [p(int)]) with Tukey post hoc pairwise comparisons. No significant differences between control and diet‐induced obese dams by pairwise comparisons. ^#^Significant difference between male and female fetuses within the same maternal dietary group (^#^ *P* < .05, pairwise comparison). Data in B, D, J and K were analysed by unpaired *t* test. Data in O were analysed by Two‐sided Fisher's Exact 2 × 2 test and showed a significantly greater number of placentas from males of diet‐induced obese dams with calcification staining >5% compared to control dams (*P* = .05; no significant effect for females)

The level of Lz protein carbonylation, an indicator of oxidative damage, was not significantly altered by diet‐induced maternal obesity (Figure 5A‐D). However, the degree of Lz malondialdehyde (MDA) abundance, which results from fatty acid peroxidation, was increased by ~25% in response to diet‐induced maternal obesity in male fetuses, but not females (Figure 5E‐G). Irrespective of dietary group, the levels of MDA in the placental Lz of males were approximately double that observed in female fetuses. There was no significant effect of maternal obesity on MDA levels in the placental Jz, although MDA scores were overall ~30% greater in the Jz of males than females regardless of the dietary group (Figure S2).

There was no impact of diet‐induced maternal obesity on the Lz abundance of activating transcription factor 5 (ATF5), which promotes cellular adaptation to stress, caseinolytic mitochondrial matrix peptidase proteolytic subunit (CLPP), chaperonin (HSP60), or tumorous imaginal disc 1/mitochondrial DnaJ co‐chaperone protein (TID1), which function in mitochondrial protein quality/unfolded protein responses (Figure 5H‐K).

Mitochondria play a role in calcium homeostasis^19^ and increased levels of calcification have been reported in the placenta of compromised pregnancies.^26^ As informed by alizarin staining (Figure 5L,M), maternal diet‐induced obesity did not affect the total area of calcification in the placental Lz (Figure 5N). However, assessment revealed that a higher number of placentas exhibited Lz calcification from males of obese dams compared to control dams (*P* = .05; Figure 5O). Calcifications tended to be located within/near large Lz sinusoids adjacent to the chorion in the males of diet‐induced obese dams. Calcification in the placental Jz was rarely observed and was unaffected by diet‐induced maternal obesity (Figure 5L,M). Thus, diet‐induced maternal obesity results in increased lipid peroxidation, calcification and cellular adaptations to stress in the placental Lz, which were all most pronounced in male fetuses.

A summary of the results of the entire study is shown in Table S2.

## 3 DISCUSSION

This study demonstrates that diet‐induced maternal obesity compromises fetal growth and placental labyrinth morphology, in association with altered levels of mitochondrial regulatory proteins, metabolic and oxidative stress pathways. The specific nature of these changes depended partly on fetal sex, whereby in male offspring relative to female offspring, diet‐induced maternal obesity exerted more pronounced changes in labyrinth structure, differentially impacted on labyrinth mitochondrial regulatory and lipid metabolic proteins, and had less of an effect on fetal weight. Moreover, fetal sex was identified as an important modifier of placental mitochondrial function, in particular in the labyrinth zone, and may provide some explanation for variations between males and females during conceptus development and with intrauterine exposures, more generally. Collectively, our data highlight that the effects of diet‐induced maternal obesity on fetal growth and subsequent offspring health may originate from sexually dimorphic changes in the placental structure and function *in utero*.

### 3.1 Maternal physiology

We used a diet high in fat and sugar to induce obesity in female mice prior to, and during, pregnancy. The composition of this diet is similar to that used in other rodent studies,^9^ and recapitulates a Western dietary pattern, high in saturated fat and simple sugar.^27^ The diet was successful in increasing maternal adiposity during pregnancy, however, the dams gained ~25% less weight during gestation and showed no significant increase in circulating leptin or adiponectin in late gestation. The reduction in pregnancy weight gain of the dam excluding the conceptuses is consistent with other studies in mice using diets to induce obesity (reviewed in [11]), and in overweight and obese women during pregnancy.^4^ In line with previous work in pregnant rodents fed obesogenic diets prior to, or just during pregnancy,^13^, ^14^, ^28^, ^29^ our diet‐induced obese dams also showed reduced circulating lipids (significant decrease in triglycerides), which likely reflect enhanced maternal storage and/or changes in feto‐placental lipid handling during gestation. The dams fed the obesogenic diet additionally showed an increase in the circulating concentration of specific pituitary hormones, with a significant elevation in LH. Previous work has shown that women who are overweight or obese, as well as female rodents fed obesogenic diets, have altered levels of LH, but the direction of change partly relates to whether other endocrine perturbations or fertility issues are also evident.^30^, ^31^, ^32^, ^33^ We did not observe any impact of diet‐induced obesity on plugging/pregnancy rates, plasma progesterone concentrations, or litter size, suggesting there were no alterations in the reproductive capacity of the mice fed the obesogenic diet. In the current study, dams showed elevated glucose, consistent with previous work indicating that diet‐induced obesity compromises maternal insulin sensitivity during gestation (recently reviewed in [7, 34]). Collectively, these studies all support the notion that obesity and/or obesogenic diets prior to, and during pregnancy, can impact maternal endocrine and metabolic state in late pregnancy.

### 3.2 Fetal development and placental labyrinth morphology

Alterations in maternal weight gain and metabolic and endocrine state in pregnancy with diet‐induced obesity were related to reduced fetal weight with fewer fetuses classified as LGA, and a corresponding increase in the proportion of SGA fetuses. These findings are consistent with previous studies in obese women reporting that reduced weight gain during gestation increases the odds of delivering an SGA infant,^4^ a subset of obese women who deliver fetal growth restricted/SGA infants,^4^, ^35^ as well as numerous animal models showing that diet‐induced maternal obesity can result in fetal growth restriction (reviewed in [5, 11, 36]). The increased incidence of SGA in our diet‐induced obese mouse dams was associated with compromised placental Lz vascularisation (fetal capillary length) and surface area for exchange. These changes in placental morphology are similar to those previously reported in obese women and non‐human primates, as well as in experimental animals fed obesogenic diets during pregnancy.^9^, ^37^, ^38^ Prior work has explored whether there are sexually dimorphic changes in fetal development in rodents and rabbits fed obesogenic diets, although whether disparities between sexes were identified, or if males or females were more adversely affected, seems to be study dependent.^17^, ^28^, ^39^ In our diet‐induced obese mouse dams, fetal growth was less affected in males than females with only a ~60% increase in SGA males compared with a figure of ~170% for the SGA females. There was also a smaller portion of LGA fetuses and a narrowing of the fetal weight range in female than male pups of diet‐induced obese dams.

Despite less severe impacts on fetal weight in males, diet‐induced maternal obesity was associated with more pronounced changes in placental Lz morphology in males compared to females, with reduced formation of the placental Lz, decreased Lz trophoblast volume, a thicker barrier to diffusion, reduced oxygen diffusing capacity and greater reduction in Lz maternal blood space volume. All of these changes would be expected to negatively affect fetal growth and development. The fetal brain to body weight ratio was increased overall by diet‐induced maternal obesity. In the males of obese dams, this may be related to the greater barrier thickness and reduced diffusion oxygen capacity seen in the placenta, as hypoxia is an established trigger for the fetal brain sparing response.^40^ The mechanism underlying changes in fetal body symmetry for the females of obese dams is unclear. Furthermore, in the placenta of females, but not males, there was an adaptive expansion of the chorion and enlargement of Lz fetal capillary diameter with maternal obesity, which would have been beneficial for Lz formation and substrate exchange capacity. Sex‐specific alterations in placental morphometric parameters have been reported for women who are overweight or obese.^41^ There are also sexually divergent effects on placental morphology in response to other suboptimal gestational environments, including maternal hypoxia, advanced age and endocrine manipulations.^17^, ^42^, ^43^, ^44^ Given the more pronounced effect of maternal obesity on placental Lz morphology, but less of an effect on fetal growth in males, these data suggest that the male sacrifices placental formation as a strategy to optimise fetal development (indeed the increase in placental efficiency in diet‐induced obese dams was most significant for males). As the placental Lz capillary diameter was larger and interhaemal membrane barrier thinner for males compared to females in control dams, perhaps male placentas have a greater intrinsic reserve capacity and are therefore, better equipped to buffer against the impact of adverse gestational environments, such as diet‐induced maternal obesity. Indeed, the sex‐associated difference in placental Lz barrier thickness was lost with maternal obesity in the current study.

### 3.3 Placental labyrinth respiratory capacity

Despite the morphological changes, mitochondrial respiratory function of the placental Lz in late gestation was largely unaffected by diet‐induced maternal obesity. There were no significant differences observed for mitochondrial OXPHOS capacity in the Lz in our diet‐induced obese dams (only moderate changes were detected by pair‐wise *t* test for FAO and FAO&CI_p_ OXPHOS in females), however, the effect of fetal sex on specific respiratory parameters in the placental Lz varied between dietary groups (FAO, electron transfer capacity, FAO coupling efficiency and complex IV enzyme activity). Our data are in contrast to other work reporting significant suppression of mitochondrial respiratory capacity for the term placenta from severely obese women with babies of normal or increased birthweight depending on the sex.^21^ Our findings also suggest that unlike other adverse gestational environments like maternal hypoxia and malnutrition,^8^, ^45^ mitochondrial respiratory capacity in the mouse placenta is largely sustained at normal levels, despite changes in placental morphological phenotype. However, sexual dimorphism was evident in Lz mitochondrial function. In particular, placental Lz samples from female fetuses presented decreased respiratory capacity with substrates for all ETS complexes when compared to those from males. However, as sex differences in Lz respiratory capacity were no longer observed after addition of rotenone (an inhibitor of complex I), our data indicate compensation of the fatty acid and NADH pathway deficiencies in Lz mitochondria for females is driven by enhanced convergence of electron transfer through the succinate pathway (electron entry into the Q junction via complex II). Indeed, when respiratory rates in the female Lz were normalized to maximal ETS capacity, they were no longer lower than those of the males, and had a higher FCR for fatty acid and complex I and II OXPHOS than seen in the male Lz. Together, these data suggest that the apparent limitation of Lz mitochondrial OXPHOS capacity observed for females is related to an overall decrease in absolute respiratory rates and electron transfer system capacity, and not to changes in the regulation patterns of OXPHOS when all respiratory substrates are present. Indeed, alterations in the use of other respiratory substrates, namely glutamine by trophoblast isolated from term human placenta is also reduced in females compared to males.^24^

Compared to females, the placental Lz of males showed lower malonate sensitivity, which confirms there is less contribution of electrons entering into the Q junction via complex II. Malonate is a competitive inhibitor of succinate dehydrogenase (SDH) and whether our finding reflects the presence of more succinate in the Lz of males (even though the samples were permeabilized) that renders them less sensitive to malonate, is yet to be determined. Regardless, alterations in the effect of malonate to inhibit Lz mitochondrial respiration, as well as the increase in Lz residual oxygen consumption, may cause males to be more prone to oxidative stress, which is consistent with the higher levels of lipid peroxidation in the Lz of males versus females (regardless of maternal dietary group). Data suggests that rates of glycolysis in the mouse placenta vary between fetal sexes during gestation.^46^ Whether diet‐induced obesity affects sex‐dependent differences in glycolytic capacity of the placenta is unknown, but this may be useful in ascertaining the mechanisms leading to increased circulating glucose levels in fetuses of these dams. Although diet‐induced obesity did not overtly affect Lz mitochondrial respiratory capacity, sex‐dependant differences appeared to be more pronounced in dams fed the obesogenic diet (eg the lower FAO, the magnitude of fatty acid complex I OXPHOS difference, ETS capacity, and complex IV activity in females versus males). Together these data identify fetal sex is an important modifier of Lz oxidative phosphorylation capacity, and sex‐differences may be amplified by diet‐induced maternal obesity.

### 3.4 Placental labyrinth mitochondrial‐related proteins

The molecular mediators that maintain mitochondrial respiration in the Lz appear differentially affected in the two fetal sexes by maternal diet‐induced obesity. Lz mitochondrial biogenesis was enhanced (indicated by PGC1A) in female, but not male offspring from obese dams. This data is in agreement with previous studies, showing that increased adiposity or obesity in the mother can be associated with altered mitochondrial biogenesis in the placenta of several species, and our study identifies that this effect could be sexually dimorphic.^47^ Enhanced Lz mitochondrial biogenesis would additionally allow any damaged mitochondria to be replenished and minimise ROS formation and oxidative stress in females of obese dams.

In males, but not females, diet‐induced maternal obesity was instead related to an up‐regulation of ETS complex II, ATP synthase and DRP1 abundance in the placental Lz. These changes may serve as a mechanism to maintain mitochondrial respiration in the face of more pronounced morphological defects in the placental Lz of males. In support of this notion, prior work has found that reductions in ETS complex abundance are linked to decreased placental mitochondrial respiration in obese women.^21^ There are also changes in the expression of mitochondrial dynamic regulators, like DRP1, that are coupled to morphological alterations in the placenta of insulin resistant rat dams,^48^ and differences in mitochondrial respiration in pig sows with increased adiposity.^47^ Akin to our findings, DRP1 is also increased in the placenta of males from diet‐induced obese rats.^49^ Up‐regulation of DRP1 would induce mitochondrial fission and fragmentation, which may have been beneficial in segregating damaged mitochondria to maintain OXPHOS capacity.^50^ However, diet‐induced maternal obesity reduced the abundance of UCP2 in the placental Lz to a similar extent in both female and male fetuses. Down‐regulation of UCP2 may have implications for ROS accumulation and cell death, which may be particularly relevant to males offspring of obese dams where calcium accumulation and lipid peroxidation were both enhanced relative to controls.

### 3.5 Placental labyrinth lipid metabolism proteins

In males, but not females, there was also enhanced inactivation of ACC (p‐ACC/total protein) in the placental Lz of diet‐induced obese dams. Enhanced ACC inactivation would reduce placental lipogenesis and favour fetal fatty acid supply, which may explain, in part, the less severe decrement in fetal growth seen in our model. In contrast, in females but not males, there was increased abundance of PPARG in the placental Lz, which would promote lipid accumulation in the obese dams. Sex differences in the handling of lipids by the placenta has been previously reported in women who are overweight/obese,^24^, ^51^ and in experimental animals fed obesogenic diets during pregnancy.^17^, ^52^ Combined these data may be informative for understanding why males born to obese mothers are more likely to be overweight as infants compared to females.^53^ Inactivation of ACC (enhanced p‐ACC) is a surrogate marker of AMPK activation, suggesting there may be an involvement of AMPK in mediating sex‐dependent alterations in lipid handling by the placenta with diet‐induced obesity, as has been suggested previously in the context of maternal diabetes.^54^ However, the Lz abundance of activated AMPK (p‐AMPK) was reduced specifically in males by diet‐induced maternal obesity, suggesting that placental energy and nutrient sensors might be dysregulated in maternal obesity and other mechanisms may be involved in the sex‐linked alterations in placental lipid metabolism and should be examined in future work. AMPK is also important for modulating Lz trophoblast differentiation and energy/cellular metabolism in mice.^55^ Hence, further work should also explore the role of sex‐dependent changes in AMPK activation in placental Lz morphogenesis, bioenergetics and metabolic function in our diet‐induced obese dams.

### 3.6 Placental labyrinth oxidative stress and signalling

Previous work has reported that diet‐induced maternal obesity can result in placental hypoxia.^56^ In the current study, we found that diet‐induced obesity resulted in increased levels of lipid peroxidation in the placental Lz of male, but not female fetuses. This increase in lipid peroxidation was associated with a higher number of males exhibiting placental calcification. An increase in the antioxidant capacity of the placenta in females,^57^ and sex differences in placental pathology that can result from in maternal obesity,^15^, ^58^ may account for the disparities in findings related to oxidative stress indices in our diet‐induced obese mice.

### 3.7 Placental junctional zone morphology and respiratory capacity

Diet‐induced maternal obesity was associated with reduced formation of the placental Jz specifically in male fetuses, although Jz spongiotrophoblast volume was decreased in both fetal sexes. However, despite these morphological changes, placental Jz respiratory capacity was unaffected by diet‐induced maternal obesity. However, future work should examine mitochondrial content and regulatory protein abundance (eg ETS complexes, dynamic regulators and stress signalling) in the Jz (as done for the Lz) to fully ascertain the effect of diet‐induced obesity on placental mitochondrial phenotype.

Placental Jz respiratory capacity in late gestation was also influenced by fetal sex. In particular, maximal OXPHOS capacity and FCR for fatty acid & complex I & II in the placental Jz were greater overall in females compared to males. Despite the fact that coupling efficiency for FAO was similar between sexes, these data are in line with previous studies in human placental tissues, showing that the female placenta presents enhanced energetic efficiency when compared to the male.^21^ Furthermore, residual oxygen consumption in the Jz was greater for males compared to females regardless of maternal dietary group, suggesting that male placentas may be subjected to increased oxidative stress as a function of oxygen consumption for oxidases and peroxidases. Indeed, lipid peroxidation was overall significantly greater in the Jz of males compared to females. As placental mitochondria consume oxygen independently of OXPHOS to synthesise steroids,^19^ sex‐dependant differences in residual oxygen consumption may have relevance for understanding variations in placental steroidogenic capacity in males versus females, which have been previously reported in humans.^16^ Future work would benefit from undertaking molecular analyses of mitochondrial regulators and functional markers in the placenta that allow direct comparisons of fetal sexes in control and diet‐induced obese dams.

### 3.8 Sex differences

The explanation for the sex‐specific effects on fetal development and placental phenotype in our control and diet‐induced obese mouse dams are unclear. However, these could originate very early, as sex defines the cellular responses of murine embryonic cells to stressors and hormones in vitro,^59^ as well as the expression of genes regulating feto‐placental formation in pre‐implantation sheep and mouse embryos exposed to maternal obesity in vivo.^60^, ^61^ Moreover, placental defects persist in mice when embryos are transferred from obese to lean mouse dams.^62^ However, whatever the mechanisms involved, sex‐dependent changes in feto‐placental phenotype identified in the current study may have relevance for understanding the disparities between male versus female offspring growth and metabolic outcomes that have been reported in women with metabolic problems, including obesity and dysglycemia,^53^ as well as in animal models fed obesogenic diets during gestation.^63^, ^64^

## 4 CONCLUSIONS

**FIGURE 6:**
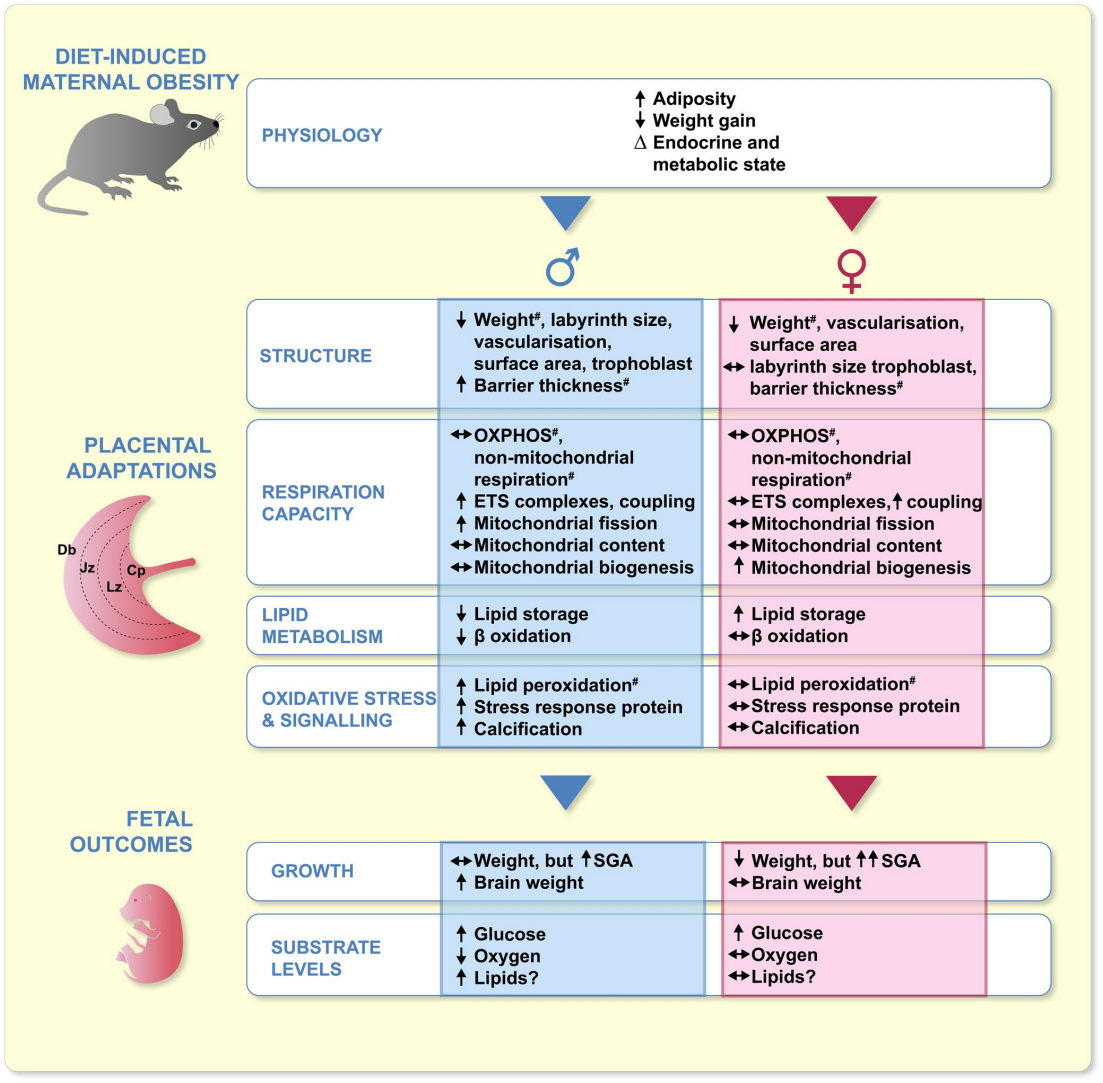
Summary of the effect of diet‐induced obesity on maternal physiology, placental phenotype and fetal outcomes with regard to fetal sex. Cp, choroid plexus; Db, decidua basalis; Jz, junctional zone; Lz, labyrinth zone; ^#^vary with fetal sex in control mice

Collectively, our data in the mouse show that diet‐induced obesity which affects the physiology of the mother during gestation, exerts sex‐linked changes in placental structure, lipid metabolism, oxidative stress, and signalling in association with alterations in fetal growth, development and substrate supply (Figure 6). As we did not include groups in which maternal physiological parameters, such as hyperglycemia and lipids are selectively altered, we were unable to identify their contribution to the changes in placental phenotype seen with the two fetal sexes in our diet‐induced obesity model. Moreover, to the best of our knowledge, there are no published in vivo studies that have looked at the effect of elevated adiposity, hyperglycemia, or altered lipids in isolation, let alone how this may be modified by fetal sex. Therefore, future work is required in this area to identify the specific mechanisms underlying the changes in placental phenotype that vary with sex, in response to maternal diet‐induced maternal obesity. Our findings may be useful in personalising therapeutic interventions offered to overweight and obese women with threatened fetal growth restriction/SGA.

## 5 MATERIAL AND METHODS

### 5.1 Animals and experimental design

Upon arrival in the animal facility, female C57BL6/J mice (7 weeks of age; Harlan) were housed under a 12:12 hour light‐dark cycle with free access to food (maintenance diet RM3, Special Diet Services) and water. After 1  week of acclimatization, mice were fed either a standard chow diet (control diet, fat: 11% kcal, simple sugars: 7% kcal; maintenance diet RM3, Special Diet Services), or a diet high in fat and sugar (obese/obesogenic diet; fat: 38% kcal, simple sugars: 33% kcal, D12451 diet, Research Diets Inc, Denmark and condensed milk) for 6 weeks prior to mating with male C57BL6/J mice (minimum 12 weeks of age). The presence of a copulatory plug was considered day 1 of pregnancy (D1). From D1, females were maintained on either the standard (n = 9) or obese diet (n = 9). Maternal weight was monitored throughout pregnancy.

### 5.2 Tissue collection and immediate maternal analyses

At D19, dams were anaesthetised (Euthatal sodium pentobarbital, Merial, Covetrus; 0.1‐0.2 mL, i.p.) and a cardiac blood sample was taken prior to schedule 1 killing by cervical dislocation. Immediately following, a mid‐line incision was made and the hysterectomised carcass and individually dissected fetuses and placentas were weighed. Fetuses were decapitated for blood collection and sexed by visual inspection of the ano‐genital distance. Placentas from fetuses that weighed closest to the mean fetal weight for each sex in the litter were manually separated into the Lz and Jz. Zones were then either immediately snap frozen or placed in cryopreservation media prior to snap freezing (0.21 M mannitol, 0.07 M sucrose, 30% DMSO, pH 7.5) and stored at −80°C for subsequent molecular and mitochondrial respiratory analyses, respectively. This cryopreservation technique has previously been shown to preserve mitochondrial respiration and structure in liver biopsies, placental samples and isolated villous trophoblasts.^65^, ^66^, ^67^ To mitigate impacts of varied placental size on placental measures, the placenta from the heaviest fetus per sex of the litter were hemisected and one half was fixed in 4% paraformaldehyde overnight before being processed and embedded into paraffin wax for stereological analyses. The percentage of viable pups per litter was calculated as the proportion of live pups minus the number of reabsorptions.

Maternal and fetal blood glucose concentrations were measured on a hand‐held glucometer (One Touch Ultra, LifeScan) and remaining maternal blood was collected into an EDTA tube then centrifuged (2,000*g* for 10 minutes). The plasma was retrieved and stored at −20°C until measurement of metabolite and hormone concentrations. Maternal fat and lean body mass contents were determined in hysterectomised pregnant mice by dual energy X‐ray absorptiometry (DEXA) scanning (Lunar PIXImus densitometer; GE Healthcare). Values were expressed as a proportion of hysterectomised weight.

### 5.3 High‐resolution respirometry

Placental mitochondrial respiratory rates were measured by high‐resolution respirometry using two substrate‐uncoupler‐inhibitor titration (SUIT) protocols (https://www.mitofit.org/index.php/SUIT‐017, ^68^). Cryopreserved Lz (n = 7‐8/sex/group) and Jz (n = 8‐9/sex/group) placental samples^65^ were gently thawed in ice‐cold sucrose solution (pH 7.5). Tissues were permeabilized in biopsy preservation medium (BIOPS; pH 7.1 solution containing 10 mM Ca‐EGTA buffer, 0.1 µM free Ca2+, 1 mM free Mg2+, 20 mM imidazole, 20 mM taurine, 50 mM K‐MES, 0.5 mM DTT, 6.56 mM MgCl2, 5.77 mM ATP and 15 mM phosphocreatine) containing saponin (5 mg/mL, Sigma‐Aldrich) for 20 minutes, followed by 3 × 5 minutes washes in respiratory medium (MiR05; pH 7.1 solution containing 20 mM HEPES, 0.46 mM EGTA, 2.1 mM free Mg2+, 90 mM K+, 10 mM Pi, 20 mM taurine, 110 mM sucrose, 60 mM lactobionate and 1 g/L BSA), all performed on ice. Following permeabilization, samples of the Lz and Jz were blotted on filter paper to determine wet tissue mass (~20 mg per region), then placed into a pre‐calibrated chamber of an Oxygraph‐2k respirometer (O2k, Oroboros Instruments). Samples were analyzed at 37°C, and oxygen concentration was kept between 200 µM and 350 µM. The DatLab software (V7, Oroboros Instruments) was used for real‐time oxygen concentration and consumption acquisition and data analysis. Respiratory rates were expressed as oxygen consumption per wet mass of placental tissue. Previous studies using cryopreserved samples report that cryopreservation does not significantly alter mitochondrial respiratory parameters, nor harm mitochondrial structure.^65^, ^66^, ^67^

Respiratory rates of the ETS were evaluated in the LEAK, OXPHOS and Electron Transfer (ET) control states. SUIT protocol 1 measured LEAK respiration (non‐phosphorylating state of intrinsic uncoupling) in the presence of malate (2 mM) and octanoyl carnitine (0.2 mM). OXPHOS capacity (phosphorylating respiration) was determined after addition of saturating concentrations of ADP (5 mM) for evaluation of FAO followed by NADH‐linked substrates pyruvate (5 mM) and glutamate (10 mM) to drive OXPHOS capacity via complex I (CI_P_). We used octanoyl carnitine, a fatty acid, as the dams were fed an obesogenic diet. Next, succinate (10 mM) was added to induce a convergent electron flow through complex I and complex II (CII_P_) into the Q‐junction, providing a measure of maximal OXPHOS capacity (FAO_&_CI_P_&CII_P_). Maximal non‐coupled ETS‐respiratory capacity was measured with titration with the protonophore, trifluoromethoxy carbonyl‐cyanide phenylhydrazone (FCCP, each dose at 0.5 µM; maximal dose 1.5 µM). Complex I inhibition by rotenone (0.5 µM) enabled the determination of the succinate‐driven respiration in the ET respiratory capacity state (CII_ETS_) and malonate (5 mM) was used to inhibit complex II. Myxothiazol (0.5 µM) and antimycin A (2.5 µM), inhibitors of complex III, provided a measure of residual (non‐mitochondrial and mitochondrial) oxygen consumption. After inhibition of respiratory complexes I, II and III, the chamber was reoxygenated and sodium ascorbate (2 mM) was added followed by N, N, N’, N’‐tetramethyl‐p‐phenylenediamine (TMPD, 0.5 mM) to assess complex IV. Complex IV activity was inhibited with sodium azide (200 mM), with the remaining oxygen flux reflecting auto‐oxidation of TMPD, as a function of oxygen pressure and TMPD, ascorbate and cytochrome *c* concentrations. The signal derived from this autooxidation was subtracted from the oxygen consumption rate measured before addition of azide to determine complex IV activity.^69^, ^70^

An additional SUIT protocol 2 was used to evaluate respiratory capacities for NADH‐linked substrates and complex II in placental Lz only, as follows: malate (2 mM) and pyruvate (5 mM) were added to measure LEAK respiration related to complex I. OXPHOS capacity was determined after addition of saturating concentrations of ADP (5 mM) and glutamate (10 mM). Next, succinate (10 mM) was added to induce a convergent electron flow through complex I and complex II (CII_P_) into the Q‐junction, providing a measure of maximal OXPHOS capacity (CI_P_&CII_P_). Subsequent additions were identical do SUIT protocol 1.

Cytochrome *c* (10 µM) was added following succinate to provide information on the integrity of the mitochondrial membranes, with data excluded if respiration increased by >30%. The average increase in respiration in the Jz was 13% and 7% for female and male fetuses, respectively; and in Lz was 14% for both female and male fetuses, irrespective of the SUIT protocol used.

The respiratory coupling states for FAO, FAO&CI_P_ and FAO&CI_P_&CII_P_ were normalized to ETS capacity (after addition of FCCP) to evaluate changes in the regulation of oxidative phosphorylation and electron transfer independent from mitochondrial content/preparation. Changes might be related to regulatory patterns of individual complexes and membrane permeability. Therefore, maximal oxygen consumption rate of each run was used as a reference state, providing a powerful normalization of flux (flux control ratio, FCR): FAO/ETS (Fatty acid oxidation FCR), FAO_&_CI_P_/ETS (fatty acid oxidation and CI FCR), FAO_&_CI_P&_CII_P_/ETS (maximal OXPHOS FCR), and CII_ETS_/ETS (CII_ETS_ FCR). Malonate sensitive respiration was calculated by subtracting oxygen consumption before and after malonate addition. OXPHOS coupling efficiency was calculated for FAO (SUIT protocol 1) for the Jz and Lz, and for the CI‐ linked substrates (SUIT protocol 2) for Lz, and represents the net OXPHOS capacity, corrected for leak respiration; 1‐ Leak/FAO and 1‐ Leak/CI_P_.

### 5.4 Morphological analyses of the placenta

#### 5.4.1 Histology and regional volume densities of placenta regions

Hemisected placentas (1 male and 1 female placenta/litter/group; Control, n = 7‐9; Obese, n = 9) were exhaustively sectioned at 7 μm perpendicular to the chorionic plate (Leica RM 2235 microtome, Leica Microsystems, Germany). Approximately 10 equally spaced sections per placenta were stained with haematoxylin and eosin and scanned (NanoZoomer digital slide scanner, Hamamatsu Photonics, UK) to assess regional volume densities of the Lz, Jz, chorionic plate and decidua using point counting as described previously.^71^ Volume densities were multiplied by placental mass to estimate the volume of each region within the placenta. All analyses were conducted blind to the group.

#### 5.4.2 Stereological analyses of the junctional zone

Morphology of the Jz was quantified using haematoxylin and eosin stained sections that were close to the mid‐line section of hemisected placentas.^44^ Briefly, volume densities of glycogen cells (GC), spongiotrophoblast (SpT), and giant cells (TGC) in the Jz were determined using point counting (16 random fields at 40× magnification using a 4 × 4 point counting grid). Values were then converted to estimated volumes by multiplying them with the estimated Jz volume for that placenta. All analyses were conducted blind to the group.

#### 5.4.3 Staining and stereological analyses of the labyrinth zone

Morphology of the Lz was revealed using double‐labelling immunohistochemistry on sections at the mid‐line section of hemisected placentas.^71^ Briefly, sections were dewaxed and rehydrated to tris‐buffered saline (TBS). Endogenous peroxidase activity was blocked with hydrogen peroxide (3% in methanol), after which antigen retrieval performed using Pepsin (0.04% in preheated 0.01 M HCl, Sigma‐Aldrich). Slides were washed in TBS, then non‐specific binding sites were blocked (dH_2_O containing 2% bovine serum albumin, 1% skimmed dry milk and 0.1% Tween20; Sigma‐Aldrich). Sections were then incubated with biotinylated lectin (isolectin B4, B‐1205, Vector Laboratories, UK; 1:250) before detection with horseradish peroxide‐conjugated streptavidin (S000‐03, Rockland Immunochemicals; 1:500), followed by 3,3′‐diaminobenzidine (Sigma‐Aldrich, UK). Sections were rinsed in water, then incubated with blocking buffer. Slides were incubated with rabbit anti‐pan cytokeratin (nb600‐579, Novus Biologicals; 1:75) at 4°C. Bound antibody was detected with alkaline phosphatase‐conjugated goat anti‐rabbit (ab6722, Abcam, UK; 1:500) followed by NBT/BCIP containing levimasole to block endogenous phosphatase (Thermofisher). Slides were washed in water and counterstained with Nuclear Fast Red (Vector Laboratories) before dehydrating and mounting with DPX (Sigma‐Aldrich). Negative control sections were prepared by omitting the primary antibodies.

Placental Lz structure was assessed in the lectin/cytokeratin‐stained sections using stereology and random systematic sampling, as described previously.^71^ Briefly, point counting (16 random fields at 40× magnification using a 4 × 4 point counting grid) was used to determine the volume densities of each Lz component (fetal capillary [FC], maternal blood space [MBS], trophoblast). The estimated volume of each component was calculated by multiplying by the estimated Lz volume. Surface densities of fetal‐ and maternal‐facing interhaemal membrane surfaces were quantified by recording the number of intersection points along cycloid arcs in 20 random fields of view at 80× magnification. These were converted to absolute surface areas by multiplying by the Lz volume. Lz fetal capillary length density, total capillary length, and diameter were obtained using counting frames (20 random fields of view at 80× magnification). Finally, the mean interhaemal membrane thickness was determined by measuring the shortest distance between FC and the closest MBS at random starting locations within the Lz, with 200 measurements made at random starting locations within the Lz per sample at 80× magnification. The values were expressed as harmonic mean interhaemal membrane thickness, which involved calculating the reciprocal of the mean of the reciprocal distances. The theoretical diffusion capacity was calculated using the total surface are (average of the FC and MBS surface areas), divided by the mean interhaemal barrier thickness and multiplied by Krogh's constant for oxygen diffusion (17.3 x 10^‐8^ cm^2^ min^−1^ kPa^−1^). The theoretical diffusion capacity was expressed relative to fetal weight to determine specific diffusing capacity.

#### 5.4.4 Placental calcification

Calcification within the placenta was assessed by Alizarin Red S staining. Briefly, paraffin embedded sections at the mid‐line of hemisected placentas (1 section/sample) were dewaxed, dehydrated to ethanol and stained with 1% Alizarin Red S solution (Sigma‐Aldrich). Slides were then washed with 100% acetone (Sigma‐Aldrich) followed by an acetone:xylene (1:1) solution. Samples were mounted in DPX (Sigma‐Aldrich) then scanned (NanoZoomer digital slide scanner, Hamamatsu Photonics). The area of calcification in the placental Lz was calculated relative to the total area of that placental region (using ImageJ, freeware). In addition, the percentage of calcification in the Lz was graded by a clinical pathologist (0%, <1%, 1%‐5%, 5%‐10%, 10%‐15% and 15%‐20%).

#### 5.4.5 Oxidative stress markers

Immunohistochemistry was performed on placental sections to detect malondialdehyde (MDA), a marker of oxidative damage to lipids arising from the peroxidation of fatty acids. Briefly, sections were dewaxed and rehydrated. Heat mediated antigen retrieval was performed using 0.01 M sodium citrate buffer (pH 6), after which endogenous peroxidase activity was blocked with hydrogen peroxide (3% in dH_2_O). Slides were washed in TBS, then non‐specific binding sites were blocked (non‐immune block: TBS containing 10% normal swine serum and 0.1% Tween20). Sections were then incubated overnight at 4°C with anti‐MDA antibody (ab6463, Abcam; 1:250 in non‐immune block). After washing sections in TBS, they were incubated for 30 minutes at room temperature with a biotinylated swine anti‐rabbit secondary antibody (E0431, Dako; 1:200 in TBS). This was followed by incubation with avidin peroxidase (5 µg/mL) for a further 30 minutes before incubating with 3,3′‐diaminobenzidine. Slides were washed in water and counterstained with haematoxylin before dehydrating and mounting with DPX (Sigma‐Aldrich). Negative control sections were prepared by omitting the primary antibodies or substituting with non‐immune rabbit IgG.

An Olympus BX41 light microscope (Southend‐on‐Sea, UK) with QIcam Fast 1394 (QImaging) at 10× magnification and Image Pro Plus 7.0 (Media Cybernetics Inc) was used to obtain images of the tissue sections. Semi‐quantitative analysis was performed by scoring each the Lz and Jz of each placental section using a scale from one to five, with a score of one equating to the lowest intensity staining and five equating to highest intensity staining.

### 5.5 Biochemical analyses

#### 5.5.1 Placental abundance of mitochondrial‐related proteins

Abundance of mitochondrial‐related proteins was determined in placental Lz samples using western blotting (n = 7/sex/group) as previously described.^72^ Briefly, total protein was extracted using RIPA buffer (Thermo Fisher Scientific) according to the manufacturer's protocol. Lysate protein concentrations were determined using a Bradford assay (Sigma‐Aldrich). Equivalent amounts of protein (60 μg) were resolved by SDS‐PAGE, blotted onto nitrocellulose (0.2 μm) and incubated with primary antibodies (Table S1). Corresponding secondary antibodies (Table S1) were applied and then visualized by enhanced chemiluminescence, SuperSignal™ West Femto Substrate (Thermo Fisher Scientific). Intensities of the bands were determined using the iBright analysis software (Thermo Fisher Scientific) and correction for protein loading was achieved by dividing the measured signal of the bands by a band of similar molecular weight on the Ponceau‐S stained membrane.

#### 5.5.2 Placental protein oxidation assay

Oxidative modification of proteins by ROS was assessed in placental Lz samples using an OxyBlot protein oxidation detection kit (Millipore) according to the manufacturer's protocol. In brief, proteins were extracted from powdered Lz tissue (60 mg) using lysis buffer. Extracted proteins (15 µg) were derivatized by 2,4‐dinitrophenylhydrazine (DNPH) treatment and then separated on a 10% polyacrylamide gel. Following electroblotting onto a nitrocellulose membrane, membrane bands were visualised using IBright imaging system (Thermo Fisher Scientific). Proteins were quantified by IBright analysis software (Thermo Fisher Scientific) and normalised to the Ponceau‐S‐stained membrane.

#### 5.5.3 Maternal hormone and metabolite concentrations

The concentrations of insulin, leptin and adiponectin in maternal plasma were measured by an enzyme‐linked immunoabsorbant assay in accordance with the manufacturers’ instructions (MesoScale Discovery, product codes K152BZC‐3, K152BYC‐2 and K152BYC‐2, respectively). The intra‐assay coefficients of variation were 6.7%, 9.3% and 7.5% for the insulin, and leptin assays, respectively. The concentrations of maternal plasma triglycerides and non‐esterified free fatty acids (NEFA) were measured by an enzymatic assay in accordance with the manufacturers’ instructions (Siemens Healthcare, USA, product code DF69A and Sigma Aldrich 11 383 175 001), with intra‐assay coefficients of variation of 5.5% and 4.5%, respectively. The concentrations of pituitary hormones, luteinizing hormone (LH), follicle stimulating hormone (FSH), thyroid stimulating hormone (TSH), growth hormone (GH), and prolactin in maternal plasma were measured by multiplex luminex kit (Millipore, product code MPTMAG‐49K) with intra‐assay coefficients of variation were 14.7%, 11.9%, 12.2%, 7.7%, and 8.2%, respectively. Finally, the concentrations of progesterone in maternal plasma were measured by an enzyme‐linked immunoabsorbant assay (Cayman Chemical, product code 582 601) with an intra‐assay coefficient of variation of 12.1%. Assays were performed by the NIHR Cambridge Biomedical Research Centre, Core Biochemistry Assay Laboratory.

### 5.6 Statistical analysis

Placental and fetal weight data were analysed by a mixed linear model with litter identified as a random effect (IBM SPSS Statistics for Windows, Version 23.0. Armonk, NY: IBM Corp). Respirometry and placental morphological analyses were performed blind to the treatment group and were analysed by two‐way ANOVA using sex and diet‐induced obesity as factors followed by Tukey's post hoc test or planned unpaired student *t* test, where appropriate (GraphPad Prism 7.0 Software). Maternal and litter characteristics, as well as Lz protein abundance were analysed by unpaired student *t* test when normally distributed or Mann‐Whitney Rank Sum test when data failed the normality or equal variance test (GraphPad Prism 7.0 Software). Shapiro‐Wilk normality tests were used to examine if data were normally distributed (GraphPad Prism 7.0 Software). Grubbs Tests were used to check for outliers in all analyses, and if identified, these were removed prior to the final statistical analysis and details of this are in any corresponding figure legends (GraphPad Prism 7.0 Software). Fetal weight frequency distribution curves and analysis were based on z‐score centiles. Two‐sided Fisher's Exact 2 × 2 tests were used to assess number of placentas showing calcification staining as <5% and >5% between groups per fetal sex (GraphPad Prism). Data are displayed as mean + SEM or mean ± SEM and were considered statistically significant at values of *P* < .05.

## CONFLICTS OF INTEREST

The authors declare that no conflicts of interest exist.

## AUTHORS’ CONTRIBUTIONS

EJC, ANSP, ALF and TEB designed the study. TN, EJC, SL, ML, MD, BB and EJM performed the experiments and TN, SL, ANSP, TEB and AM analyzed and graphed the data. ANSP wrote the paper in consultation with EJC, ALF and TEB. All authors contributed to data interpretation and performed final editing checks and approved the final manuscript.

## ETHICAL APPROVAL

This study was performed in accordance with Home Office regulations under the UK Animals (Scientific Procedures) Act 1986 and was approved by the Ethical Review Committee of the University of Cambridge (project licence number PC6CEFE59). All the material submitted conforms with good publishing practice in physiology, including following the 3Rs principles.^73^

## Supporting information

Fig S1

Fig S2

Tables S1‐S2

## Data Availability

The data that support the findings of this study are available from the corresponding authors upon reasonable request.
