# Supplementary figures and images for: Diet‐induced maternal obesity impacts feto‐placental growth and induces sex‐specific alterations in placental morphology, mitochondrial bioenergetics, dynamics, lipid metabolism and oxidative stress in mice
Source: Acta Physiol (Oxf). 2022 Feb 15;234(4):e13795. doi: 10.1111/apha.13795 (PMC9286839; doi:10.1111/apha.13795)

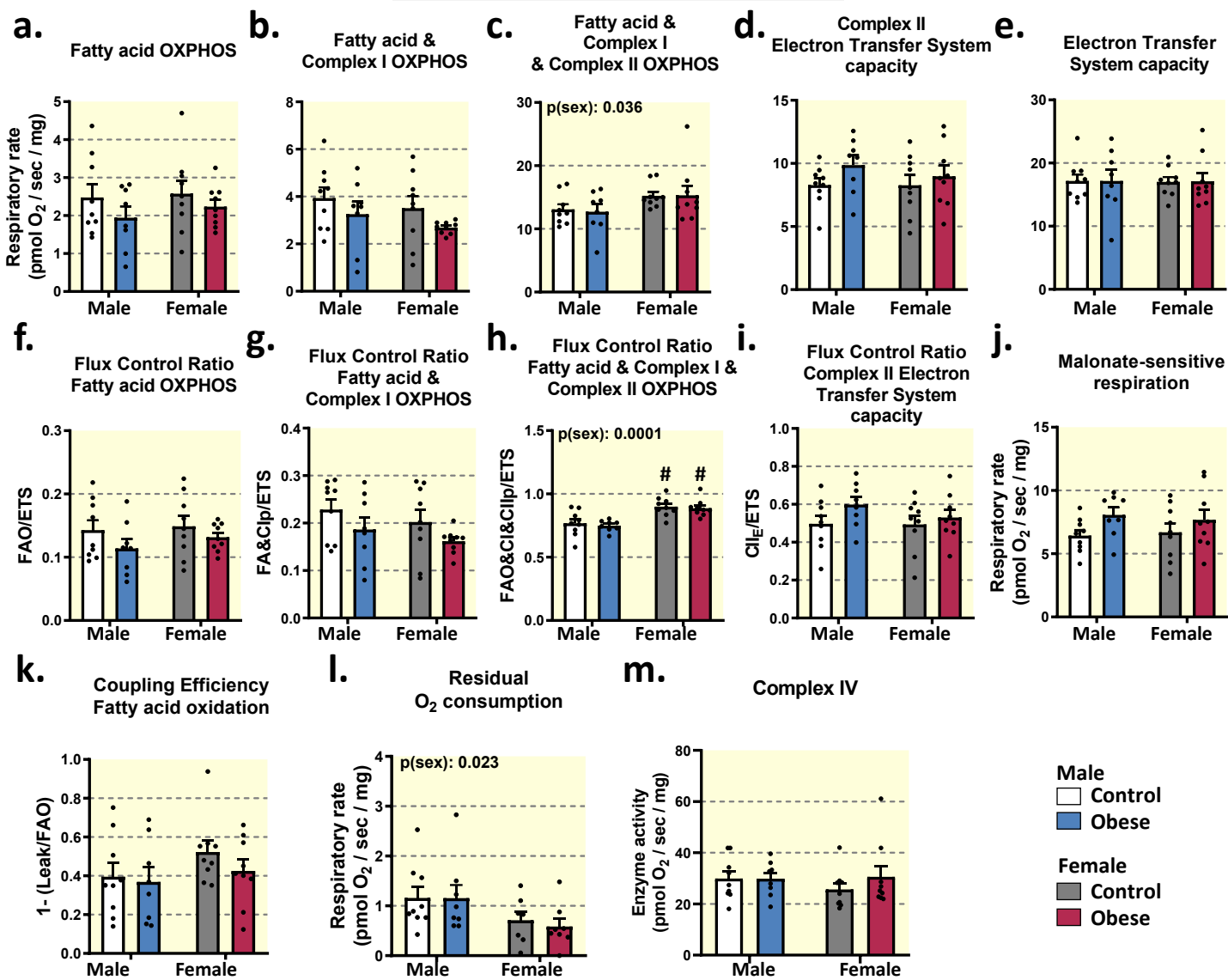

Supplement: Supplementary file 1 — Fig S1 [file APHA-234-0-s003.pdf]

a.

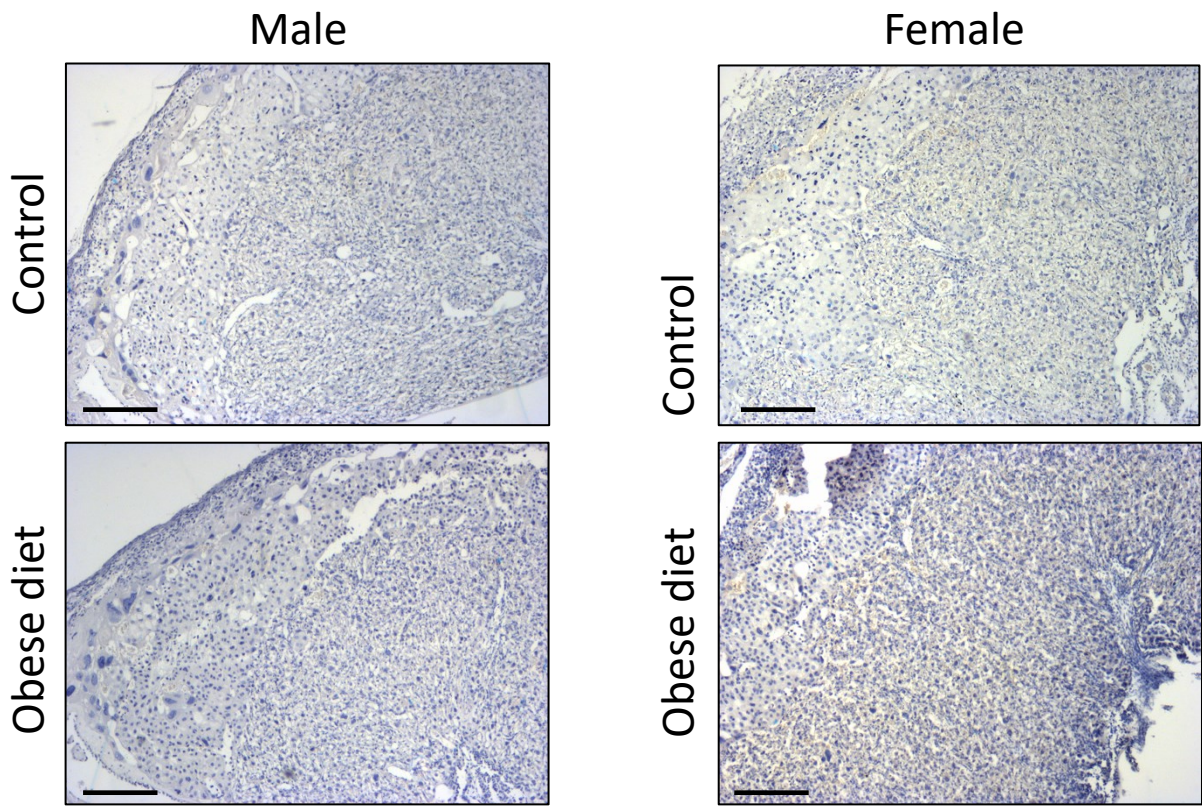

b.

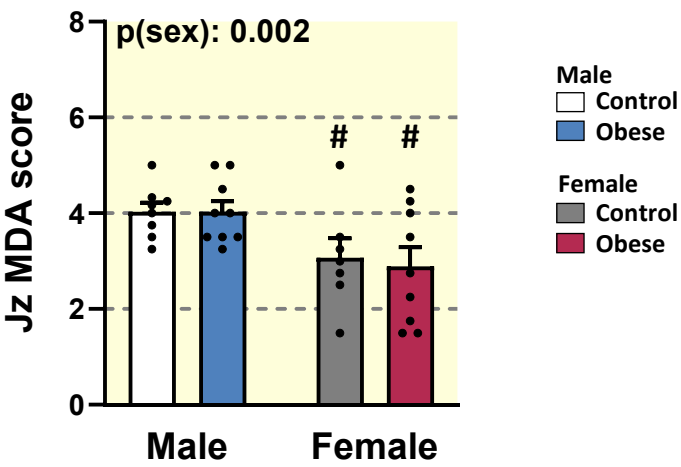

Supplement: Supplementary file 2 — Fig S2 [file APHA-234-0-s002.pdf]
