# Supplementary material for: Diet‐induced maternal obesity impacts feto‐placental growth and induces sex‐specific alterations in placental morphology, mitochondrial bioenergetics, dynamics, lipid metabolism and oxidative stress in mice
Source: Acta Physiol (Oxf). 2022 Feb 15;234(4):e13795. doi: 10.1111/apha.13795 (PMC9286839; doi:10.1111/apha.13795)
Supplement: Supplementary file 3 — Tables S1‐S2 [file APHA-234-0-s001.docx]

**Supplementary Tables**

**Supplementary Table 1. Details of the antibodies used for western blotting.**

| **Protein of interest** | **Species** | **Company** | **Catalogue #** | **Dilution** |
| --- | --- | --- | --- | --- |
| CLPP | Rabbit | Abcam | ab124822 | 1:1000 |
| TID1 | Rabbit | GeneTex | GTX111077 | 1:1000 |
| HSP60 | Rabbit | Abcam | ab46798 | 1:1000 |
| ETS complexes | Mouse | Life Technologies | 458099 | 1:1000 |
| Citrate synthase | Rabbit | Abcam | ab96600 | 1:1000 |
| DRP1 | Rabbit | CST | 8570 | 1:1000 |
| MFN2 | Rabbit | CST | 9482 | 1:1000 |
| OPA1 | Rabbit | CST | 80471 | 1:1000 |
| ACC | Rabbit | CST | 3676 | 1:1000 |
| pACC (site: Ser79) | Rabbit | CST | 3661 | 1:1000 |
| ATF5 | Rabbit | Abcam | ab184923 | 1:1000 |
| PGC1A | Rabbit | Santa Cruz | SC-13067 | 1:500 |
| PPARG | Mouse | Santa Cruz | SC-7273 | 1:200 |
| UCP2 | Rabbit | Biolegend | #615902 | 1:500 |
| AMPK | Rabbit | CST | 2532 | 1:1000 |
| pAMPK (site: Thr172) | Rabbit | CST | 2531 | 1:1000 |
| Amersham ECL Rabbit IgG, HRP-linked | Donkey | Cytiva | NA934 | 1:10000 |
| Amersham ECL Mouse IgG, HRP-linked | Sheep | Cytiva | NA931 | 1:10000 |

Abcam, UK; CST, Cell signalling technologies, USA; Cytiva, USA; Biolegend, USA, ETS, electron transfer system, GeneTex, USA; Life Technologies, USA.

**Supplementary Table 2. Summary of the effect of diet-induced obesity on feto-placental phenotype and the influence of fetal sex.**

| **Parameter** | **Males in OB *vs* CT** | **Females in OB *vs* CT** | **Female *vs* males in CT or OB** |
| --- | --- | --- | --- |
| **Fetal weight** | ↔(↓6%) and ↑61% SGA | ↓9% and ↑172% SGA | NS |
| **Placental weight** | ↓24% | ↓22% | M>F in CT not OB |
| **Jz size** | ↓30% | ↔(NS: ↓3%) | M>F in CT not OB |
| **SpT volume** | ↓32% | ↓18% | ↔ |
| **Chorion size** | ↔(NS: ↑5%) | ↑177% | NS |
| **Lz size** | ↓26% | ↔(NS: ↓15%) | NS |
| **Lz T volume** | ↓39% | ↔ (NS: ↓23%) | NS |
| **Lz MBS volume** | ↓25% | ↓14% | NS |
| **FC volume** | ↔ | ↔ | M>F in CT and OB |
| **MBS surface area** | ↓30% | ↓33% | NS |
| **FC surface area** | ↓24% | ↓25% | NS |
| **FC length** | ↓24% | ↓30% | NS |
| **FC capillary** | ↑14% | ↑13% | M>F in OB not CT |
| **Barrier thickness** | ↑35% | ↔(NS: ↑5%) | M<F in CT not OB |
| **Specific diffusing capacity** | ↓43% | ↔(NS: ↓27%) | NS |
|  |  |  |  |
| **Jz FA OXPHOS** | ↔ | ↔ | NS |
| **Jz FA&CI OXPHOS** | ↔ | ↔ | NS |
| **Jz FA&CI&CII OXPHOS** | ↔ | ↔ | M<F in CT and OB |
| **Jz CII ETS** | ↔ | ↔ | NS |
| **Jz ETS** | ↔ | ↔ | NS |
| **Jz FCR FA** | ↔ | ↔ | NS |
| **Jz FCR FA&CI OXPHOS** | ↔ | ↔ | NS |
| **Jz FCR FA&CI&CII OXPHOS** | ↔ | ↔ | M<F in CT and OB |
| **Jz FCR CII ETS** | ↔ | ↔ | NS |
| **Jz malonate-sensitive respiration** | ↔ | ↔ | NS |
| **Lz FA OXPHOS** | ↔ | ↔ | M>F in OB not CT |
| **Lz FA&CI OXPHOS** | ↔ | ↔ | M>F in OB not CT |
| **Lz FA&CI&CII OXPHOS** | ↔ | ↔ | M>F in CT and OB |
| **Lz CII ETS** | ↔ | ↔ | NS |
| **Lz ETS** | ↔ | ↔ | M>F in OB not CT |
| **Lz FCR FA** | ↔ | ↔ | NS |
| **Lz FCR FA&CI OXPHOS** | ↔ | ↔ | NS |
| **Lz FCR FA&CI&CII OXPHOS** | ↔ | ↔ | M<F in CT and OB |
| **Lz FCR CII ETS** | ↔ | ↔ | M<F in CT and OB |
| **Lz malonate-sensitive respiration** | ↔ | ↔ | M<F in CT and OB |
| **Lz ETS complexes and UCP2** | CII: ↑63%, ATPase:↑38%  UCP2: ↓68% | ↔ (NS CI: ↑8%, ATPase:↑37%)  UCP2: ↓44% | ND |
| **Lz CS** | ↔ | ↔ | ND |
| **Lz mitochondrial dynamics** | DRP1: ↑74%  PGC1A ↔ (NS: ↑12%) | ↔ (NS: ↑35%)  PGC1A: ↑74% | ND |
| **Lz lipid handling and AMPK** | pACC/Total: ↑98%  PPARG: ↔ (NS: ↑10%)  pAMPK: ↓49% | pACC/Total: ↔ (NS: ↑5%)  PPARG: ↑291%  pAMPK: NS (↑5%) | ND |
| **Lz MDA** | ↑25% | ↔ (NS: ↑27%) | M>F in CT and OB |
| **Lz ATF5** | ↔ | ↔ | ND |

CT = control dams, ND = not-determined, NS= not-significant, OB = diet-induced obese dams, vs = versus. Arrows indicate direction of change and ↔ indicates no change.

ACC, acetyl-CoA carboxylase; AMPK, 5' AMP-activated protein kinase; ATPase, ATP synthase; BMI, body mass index; CI_P_, OXPHOS capacity via complex I; CII_P_, OXPHOS capacity via complex II; DRP1, dynamin related protein; ETS, electron transport system; FAO, fatty acid oxidation; FC, fetal capillaries; FCR, flux control ratio; Jz, junctional zone; Lz labyrinth zone; MBS, maternal blood spaces; MDA, malondialdehyde; OXPHOS, oxidative phosphorylation; PGC1A, peroxisome proliferator-activated receptor gamma coactivator 1-α; PPARG, peroxisome proliferator activated receptor-γ, SGA, small for gestational age; SpT, spongiotrophoblast; UCP2, uncoupling protein-2.

**Supplementary figures**

**Supplementary Figure 1. Placental junctional zone respiratory capacity in control dams and the impact of diet-induced obesity.** Mitochondrial respiration in the placental junctional zone **(a-m).**  **a-c** corresponds to fatty acid, complex I and complex II substrate-driven respiration; **d** corresponds to complex II-driven oxygen consumption after addition of rotenone; **e** corresponds to maximal electron transfer system (ETS) capacity after addition of FCCP; **f-i** correspond to oxygen consumption for the respective substrates relative to ETS capacity; **j** corresponds to the fraction of oxygen consumption that was inhibited by malonate. Data in **k,** correspond to coupling efficiency after addition of the stated respiratory substrate calculated as 1- (Leak/Coupled respiration) where Leak is the oxygen consumption after addition of malate and octanoylcarnitine; **l** corresponds to oxygen consumption not related to mitochondrial complexes, calculated after addition malonate, antimycin A and myxothiazol; **m** corresponds to the enzymatic assay of complex IV activity, measured in the same run as the respiratory states using the artificial substrate TMPD and ascorbate. Data are displayed as individual values with mean + SEM with 1 fetus per sex in each of 7-9 litters per group, and were analysed by two-way ANOVA (fetal sex [p(sex)], diet-induced maternal obesity [p(diet)], interaction [p(int)]) with Tukey *post hoc* pairwise comparisons. No significant differences between control and diet-induced obese dams by pairwise comparisons. # Significant difference between male and female fetuses (#P<0.05).

**Supplementary Figure 2.** **Malondialdehyde (MDA) staining. a**) Representative negative control sections for MDA staining of mouse placentas and **b)** and quantification of MDA staining in the junctional zone. Scale bars in **a** on the lower magnification images are 250µm. Data in **b** are from 7 litters per group, with 1 pup per sex in each litter and displayed as individual values with means ± SEM and analysed by two-way ANOVA (fetal sex [p(sex)], diet-induced maternal obesity [p(diet)], interaction [p(int)]) with Tukey *post hoc* pairwise comparisons. No significant differences between control and diet-induced obese dams by pairwise comparisons. # Significant difference between male and female fetuses (#P<0.05, pairwise comparison).
